# Proteometabolomic Study of Compatible Interaction in Tomato Fruit Challenged with *Sclerotinia rolfsii* Illustrates Novel Protein Network during Disease Progression

**DOI:** 10.3389/fpls.2016.01034

**Published:** 2016-07-26

**Authors:** Sudip Ghosh, Kanika Narula, Arunima Sinha, Rajgourab Ghosh, Priyanka Jawa, Niranjan Chakraborty, Subhra Chakraborty

**Affiliations:** National Institute of Plant Genome ResearchNew Delhi, India

**Keywords:** 2-DE coupled mass spectrometry, comparative proteomics, metabolite profiling, patho-stress, protein network, *Sclerotinia*, tomato fruit

## Abstract

Fruit is an assimilator of metabolites, nutrients, and signaling molecules, thus considered as potential target for pathogen attack. In response to patho-stress, such as fungal invasion, plants reorganize their proteome, and reconfigure their physiology in the infected organ. This remodeling is coordinated by a poorly understood signal transduction network, hormonal cascades, and metabolite reallocation. The aim of the study was to explore organ-based proteomic alterations in the susceptibility of heterotrophic fruit to necrotrophic fungal attack. We conducted time-series protein profiling of *Sclerotinia rolfsii* invaded tomato (*Solanum lycopersicum*) fruit. The differential display of proteome revealed 216 patho-stress responsive proteins (PSRPs) that change their abundance by more than 2.5-fold. Mass spectrometric analyses led to the identification of 56 PSRPs presumably involved in disease progression; regulating diverse functions viz. metabolism, signaling, redox homeostasis, transport, stress-response, protein folding, modification and degradation, development. Metabolome study indicated differential regulation of organic acid, amino acids, and carbohydrates paralleling with the proteomics analysis. Further, we interrogated the proteome data using network analysis that identified two significant functional protein hubs centered around malate dehydrogenase, T-complex protein 1 subunit gamma, and ATP synthase beta. This study reports, for the first-time, kinetically controlled patho-stress responsive protein network during post-harvest storage in a sink tissue, particularly fruit and constitute the basis toward understanding the onset and context of disease signaling and metabolic pathway alterations. The network representation may facilitate the prioritization of candidate proteins for quality improvement in storage organ.

## Introduction

Cross-talk between pathogens and plants originates from a co-evolution of invasion and defense strategies (Chisholm et al., 2006). Plants respond to pathogens by deploying a sophisticated array of multifaceted rapid and specific reactions (Benhamou, 1996). Events in the plant-pathogen interaction either culminate in resistance or susceptibility. However, not all organs respond equally to invading pathogens, and susceptibility can depend on developmental state (Cantu et al., 2009). Generally, patho-stress is the major impediments in post-harvest storage, which not only deteriorates the fruit nutritional quality and sensorial attributes, but also shelf-life. Tomato, the second most consumed vegetable of high economic value represents a model for plant development, fleshy fruit physiology, ripening, and pathology (Arie et al., 2007; Giovannoni, 2007). It is an assimilator of various metabolites thus appears to be the superlative target for pathogen attack.

Through convergent and divergent adaptations, phyto-pathogenic fungi have evolved diverse lifestyles, ranging from obligate biotrophs to necrotrophs, and from host-specific to broad host range. Necrotropic fungi, including *Sclerotinia sp*. are the largest class of fungal pathogens and cause serious crop losses worldwide. It is the causal agent of stem rot and the most common non-host ascomycetes fungal pathogen capable of infecting over 400 plant species primarily dicotyledonous herbs from 278 genera and 75 families, including tomato (Kwon and Park, 2002). Typical visible disease symptoms include appearance of white silky and thread-like mycelia on the fruits. Under environmental conditions conducive to germination, growth and spread, infection can result in devastating yield and post-harvest loss. Attempts have been made to understand the *Sclerotinia* sp. pathogenicity, but it is still a difficult pathogen to manage, control, and eradicate (Mullen, 2001).

There is no known tomato resistant cultivar against *Sclerotinia* rot (Abdeljalil et al., 2016). It has also been shown that *Arabidopsis, Phaseolus*, and sunflower are susceptible to *Sclerotinia* infection and no resistant cultivars are known, with only exception being *Brassica* (van Becelaere and Miller, 2004; Perchepied et al., 2010; Garg et al., 2013; Oliveira et al., 2015). Thus, to understand the infection strategy of necrotrophic fungi, *Sclerotinia* in particular, screening of differential proteome and transcriptome is an important consideration (Liang et al., 2008; Garg et al., 2013; Oliveira et al., 2015). Capturing the differential proteome upon *Sclerotinia* infection for a susceptible host would help identifying possible host factor and virulence determinant. Furthermore, network analysis often led to the identification of potential targets that may be essential for understanding disease mechanism (Cantu et al., 2009; Chi et al., 2013). Reverse genetics approach using mutants, RNAi, and Crisper may target such host factors to silence the candidate gene/protein thereby imparting resistance (May et al., 2005; Bonaldi et al., 2008; Katiyar and Jin, 2010; Tinoco et al., 2010; Cary et al., 2011; Younis et al., 2014; Mishra et al., 2016). Targeting such host proteins for making the crop tolerant to *Scleroninia* infection may benefit breeding/quality improvement program preventing fruit spoilage during storage for post-harvest management of tomato (Jnra et al., 2000). Despite many studies concerning the regulation of host biochemical machinery by *Sclerotinia* causing destructive pathogenesis resulting in extensive necrosis (Garg et al., 2010), knowledge about the multifactorial interaction and translational reprograming in plants during such compatible interaction remains largely unknown.

Translational reprogramming might play an important role in determining diseased vs. immune state during host-pathogen interaction. The effect of disease processes on cellular protein networks and function are of crucial importance in improving plant tolerance levels against patho-stress. In recent years, proteomic approach became increasingly important to study translational reprograming (Aebersold and Cravatt, 2002). Most of the functional proteomic studies have used different organs of tomato (Rocco et al., 2006; Sheoran et al., 2007; Barsan et al., 2010; Page et al., 2010; Chakraborty et al., 2013; Manaa et al., 2013). Considerable advances have been made in understanding disease development using differential proteomic studies in response to fungi, virus, herbivory, and nutrient deficiency disease blossom end rot (Casado-Vela et al., 2005, 2006; Shah et al., 2012). To facilitate the interpretation, time-series experiments are often employed to capture dynamic expression profiles that distinguish primary from secondary response in protein regulatory networks (Huang and Fraenkel, 2009). However, the temporal kinetics of the proteome and the protein network in host response during compatible interaction in fungal disease has remained unexplored. Elucidating fungal target host proteins is thus necessary to understand how they act to subvert plant immune responses.

Here, we report, the comparative proteome analysis of tomato fruit infected with *Sclerotinia rolfsii*. Attention was focused to investigate the dynamic nature of the protein network in relation to patho-stress. We developed for the first time temporal profiling of proteins in a sink organ challenged with a necrotroph. This study provides a detailed framework of fruit protein patterning and proteins involved in signaling, transcription regulation, and metabolite reallocation during fungal invasion and subsequent disease development. The pathways identified by comparative proteomics were validated by analyzing the metabolome. Data integration achieved through network study revealed numerous correlations involved in regulatory processes, which may give new insights into the disease progression.

## Materials and methods

### Plant material and experimental design of patho-stress

Tomato *var*. Pusa Ruby was grown in an experimental field of National Institute of Plant Genome Research during October. In brief, seeds were sown in a mixture of soil:vermiculite (3:1 [v/v]) and seedlings were transplanted to commercial tomato-cultivated soil at 3–4 leaves stage. Equivalent-sized red ripe fruits (4–5 days past color break) were harvested by criteria previously described (Rose et al., 1997; Catala et al., 2011) from three randomized plots. Each biological replicate consisted of nine fruits of the same stage from three different plants.

One mm potato dextrose agar (PDA) plugs containing actively growing *S. rolfsii* mycelia were used as inoculum. The freshly harvested whole fruits were infected at the stylar region and were incubated at 22°C under high humidity to allow disease progression upto 120 h post-inoculation (hpi). Unstressed control fruits were inoculated only with PDA plugs under identical conditions. To ensure the profiling of host proteins, tissues from patho-stressed fruits were collected at different time points (24, 48, 72, 96, and 120 hpi) after removal of fungal mycelium. The unstressed fruit samples were collected at same time points and finally pooled to normalize the storage effects, if any. Tissues were immediately frozen in liquid nitrogen, ground to a fine powder, and stored at −80°C until further use.

### Fruit protein extraction, 2-DE, and image analysis

Soluble proteins were isolated from patho-stressed and unstressed tomato fruits according to previously published method (Chakraborty et al., 2013). In brief, 2.5 g of frozen whole fruit tissue powder was homogenized in three volumes of extraction buffer containing 700 mM sucrose, 500 mM Tris-HCL pH 7.5, 100 mM KCL, 50 mM EDTA, 2% [v/v] β-mercaptoethanol and 1 mM PMSF by vortexing 15 min on ice. The total proteins were recovered by phenol extraction method. The mixture was vortexed for 10 min and centrifuged at 10,000 g at 4°C and the soluble proteins were recovered as upper phenol phase. Proteins were then precipitated by addition of five volume of 100 mM ammonium acetate in methanol overnight at –20°C. Precipitated proteins were centrifuged at 10,000 g for 30 min and the protein pellets were washed once with ice-cold methanol and three times with ice-cold acetone, air dried and resuspended in 2-D rehydration buffer.

Protein concentration was determined by the 2-D Quant kit (GE Healthcare). Protein samples (300 μg) were loaded onto IPG strips (Immobiline DryStrip pH 4–7 NL, 13 cm; GE Healthcare Biosciences) by in-gel rehydration, and isoelectric focusing was carried out using IPGphor system (Amersham Biosciences, Bucks, U.K.) at 20°C for 35,000 Vh with current limit set to 50 μA/strip. The focused strips were subjected to reduction with 1% (w/v) DTT in 10 mL of equilibration buffer [6 M urea, 50 mM Tris-HCl (pH 8.8), 30% (v/v) glycerol and 2% (w/v) SDS], followed by alkylation with 2.5% (w/v) iodoacetamide in the same buffer. The strips were then loaded on top of 12.5% SDS-PAGE for second dimension separation (Chakraborty et al., 2013). Gels were stained with Silver Stain Plus kit (Bio-Rad) and scanned with a Bio-Rad FlourS system.

Gel images were analyzed with PDQuest 7.2.0 (Bio-Rad). For each time point three 2-DE gels representing three biological replicates were used for the data analysis (Supplementary Figure S1). The correlation coefficient has been maintained to at least 0.8 between the replicate gels (Supplementary Figure S2). The detailed data analyses were carried out as described previously (Chakraborty et al., 2013). To compare spots across gels, a matchset representing a “standard image” of three replicates was created from six time points. Low quality spots (<30 quality score) were removed from further analysis. Next, for comparison, protein spots observed in each time point were normalized to “total density in gel image” mode and spots were manually annotated. The spot volumes were further normalized using three unaltered protein spots across all the gels. The average normalized quantity, SD, and CV of identified differentially expressed spots are provided in Supplementary Table S1. The spots considered differentially abundant if present either in three diseased state or in two diseased state and untreated control. The significantly altered (Log_2_ > 1.32, *p* < 0.05) spots with more than 2.5-fold change in abundance were selected for identification (Supplementary Table S2). All statistical analyses were performed as explained in the “Statistical Analysis” Section.

### Protein digestion and MS analysis

The protein spots were mechanically excised from the gels, destained, and trypsin-digested prior to MS analysis according to standard technique (Casey et al., 2005). A total of 74 trypsinolyzed protein spots were loaded onto a C_18_PepMap100 column (3 μm, 100 Å, 75 micron ID_15 cm) at 300 nL/min (LCPackings) and separated with a linear gradient of water/acetonitrile/0.1% formic acid (v/v) and analyzed by electrospray ionization using an ultimate 3000 nano HPLC system (Dionex) coupled to a 4000 Q-TRAP mass spectrometer (Applied Biosystems). The peptides were eluted with a gradient of 10–40% acetonitrile (0.1% formic acid) over 60 min. Eluted peptides were electrosprayed into the mass spectrometer operated in positive mode and peptide analysis was performed using data-dependent acquisition of MS scan (m/z from 400 to 1800) followed by MS/MS scans. The MS/MS data were extracted using Analyst software version 1.5.1 (Applied Biosystems). The detailed analysis was performed as described previously (Chakraborty et al., 2013).

Nineteen digested protein spots were analyzed through 4800 MALDI-TOF/TOF analyzer (Applied Biosystems). The α-cyano-4-hydroxycinnamic acid (CHCA) matrix was prepared at one-half saturation in acetonitrile/water 1/1 (v/v) acidified with 0.1% TFA. A 1 μL aliquot of each sample mixed with an equal volume of matrix solution was immediately spotted onto the MALDI target plate and allowed to dry at room temperature. The reflected spectra were obtained over a mass range of 850–4000 Da. The spectra of 100 laser shots were summed to generate a PMF for each protein digest. Suitable precursors for MS/MS sequencing analyses were selected, and fragmentation was carried out using collision-induced dissociation (CID; atmospheric gas was used) in 1 kV ion reflector mode and precursor mass windows of +5 Da.

### Database searching for protein identification, functional annotation, and expression clustering

The m/z spectra were searched against a target database created by combining the *S. sclerotiorum* protein database (http://www.broadinstitute.org/annotation/genome/sclerotinia_sclerotiorum/MultiDownloads.html; 14503 sequences) with the SGN Tomato database ITAG 2.3 release (34727 sequences, 11956401 residues) available at http://solgenomics.net/organism/Solanum_lycopersicum/genome using the Mascot v.2.1 (http://www.matrixscience.com) search engine. For LC-MS/MS analysis peak lists were searched against the combined target database supplemented with contaminant database using the MASCOT v.2.1 (http://www.matrixsciences.com) search engine analysis. The database search criteria were: taxonomy, all entries; peptide tolerance, ±1.2 Da; MS/MS tolerance, ±0.6 Da; peptide charge +1 +2 or +3; maximum allowed missed cleavage, 1; fixed modification, cysteine carbamidomethylation; variable modifications, methionine oxidation; instrument type, ESI-TRAP. The score threshold to achieve *p* < 0.05 is set by Mascot algorithm and is based on the size of the database used in the search. We considered only those proteins whose MOWSE score was above the significant threshold level. For MALDI-TOF/TOF analysis, the search for peptides was performed using GPS explorer v 3.6 software (Applied Biosystems) with MASCOT algorithm with the following search parameter: digestion enzyme/trypsin with one missed cleavage; fixed modification, cysteine carbamidomethylation; and variable modification, methionine oxidation; MS (precursor-ion) peak filtering: monoisotopic, minimum S/N 10, mass tolerance ± 100 ppm; MS/MS (fragment-ion) peak filtering: monoisotopic, minimum S/N = 3, MS/MS fragment tolerance ± 0.4 Da. Proteins with C.I.% > 95% were considered as a positive identification and were also evaluated on the basis of various parameters such as number of peptides matched, MOWSE score, quality of the peptide maps, percent coverage of the matched protein, besides similarity of theoretical and experimental protein molecular masses. The significance threshold was set to *p* < 0.05 and false discovery rate (FDR) < 0.05 for the Mascot search. For the number of observed peptides per protein, the unique sequences were counted and were imported to Excel spreadsheets (Supplementary Table S3). The abundance of each identified protein was estimated by determining the protein abundance index (PAI) and the emPAI. The corresponding protein content in mol% was calculated as described previously (Ishihama et al., 2005; Supplementary Table S4).

The protein functions were assigned using a protein function database, Pfam or InterPro. The identified proteins were divided into different functional classes according to gene ontology (GO) and literature. BLASTP search of identified protein sequences was performed through Blast2GO (Conesa et al., 2005) against Uniprot protein database with a minimum expectation value of 1 × 10^−3^. Annotations were retrieved with default parameters: pre-*e*Value-Hit-Filter at 1 × 10^−6^, cut-off was set at 55 and GO weight at 5. Self-organizing tree algorithm (SOTA) clustering was performed on the log-transformed fold induction expression values across time points using Multi Experiment Viewer (MeV) software (Saeed et al., 2003). The clustering was done with the pearson correlation as distance with 10 cycles and a maximum cell diversity of 0.8 (Romijn et al., 2005). *P* < 0.05 were considered statistically significant.

### Western blot analysis

Immunoblotting was carried out with 50 μg protein on 12.5% SDS-PAGE. The electrophoresis was performed at room temperature and the proteins were electroblotted onto Hybond-C membrane (Amersham Biosciences, U.K.) at 150 mA for 2 h. The membranes were blocked with 5% (w/v) nonfat milk in TBST buffer (0.1 M Tris pH 7.9, 0.15 M NaCl and 0.1% Tween 20) and probed with primary polyclonal anti-MSR (ab16803) and anti-DnaK (ab80161) (Abcam Ltd., U.K.) at varying dilutions (1:1000–1:15000) in TBS buffer. Immunodetection was performed with horse radish peroxidase conjugated anti-goat IgG (Abcam Ltd., U.K.) as secondary antibody at a concentration of 1:20000 for detection using enhanced chemiluminescence (Pierce). X-ray film from immunoblotting was scanned using Bio-Rad FlourS system equipped with a 12-bit camera and exported in tiff format. Protein quantification was performed using the Fluor S MultiImager (Bio-Rad) and Quantity 1-D Analysis software (Bio-Rad) using the volume analysis function, and the relative signals were calculated.

### Primer design and qRT-PCR analysis

Five key PSRPs were selected from the *S. rolfsii* infected tomato fruit proteome for a follow-up study of gene expression by qRT-PCR analysis. The nucleotide sequences of the corresponding proteins ware obtained by performing a TBLASTN search in SGN tomato database. For each candidate gene, primers were designed using Primer Express v3.0 software (Applied Biosystems). Primer sequences are listed in Supplementary Table S5.

Total RNA from unstressed and infected (24–120 hpi) tomato fruits were isolated using RNeasy Plant Mini Kit (Qiagen). The reverse transcription was carried out with 2 μg of RNA using SuperScript VILO cDNA Synthesis Kit (Invitrogen). The qRT-PCR was performed in three biological and three technical replicates with the ABI PRISM 7700 Sequence Detection System (Applied Biosystems) using SYBR Green PCR Master Mix and gene-specific primer pairs in a final volume of 20 μl, including cDNA template. 18s rRNA is used as endogenous control for normalizing the qRT-PCR data and relative quantification (2^−ΔΔCt^). Expression ratios of mRNA transcripts at 24, 48, 72, 96, and 120 hpi relative to control (0 hpi) were calculated and statistically tested.

### Isolation, extraction, derivatization, and GC-MS analysis of fruit metabolites

For metabolite analyses, unstressed, and patho-stressed (120 hpi) red ripe tomato fruit were used according to previously published method (Agrawal et al., 2013). The experiments were performed at least in four replicates. Metabolites were extracted and derivatized as described by Schauer et al. (2005). In brief, 350 mg fruits were homogenized in 1400 μl 100% methanol with 50 μl ribitol as internal standard (2 mg ml^−1^) and extracted for 15 min at 70°C. The extract was mixed with one volume water and centrifuged at 2200 g. Subsequently, methanol/water supernatant was aliquoted to 1 ml and dried in vacuo for 9–16 h. The dried residue was re-dissolved and derivatized using 80 μl of 20 mg ml^−1^ methoxyamine hydrochloride in pyridine for 90 min at 30°C followed by a 30 min treatment of 80 μl MSTFA at 37°C. Forty microliter of retention time standard mixture was added prior to trimethylsilylation.

The derivatized extracts were diluted 10-folds in n-heptane and a sample volume of 1 μl was injected in splitless mode into Shimadzu GCMS-QP 2010 plus. The mass spectrometer was tuned according to the manufacturer's recommendations. GC was performed on an Rtx5MS-30 m column with 0.25 mm I.D. and df 0.25 (Restek). The injection temperature was set at 260°C, interface was set at 270°C, and ion source was adjusted to 230°C. Helium was used as the carrier gas at a flow rate of 1 mL min^−1^. The analysis was performed using the temperature program described in Schauer et al. (2005). Mass spectra were recorded at 2 scan s^−1^ with an m/z 40 to 600 scanning range. Peaks were assigned, quantified, and all data were normalized to the mean response calculated for the control of each replicate; to allow comparison between the samples, individual values were normalized in the same way as per Roessner et al. (2000). The recovery of small representative amounts of each metabolite through the extraction, derivatization, storage, and quantification procedures has been followed and documented as detailed previously. Targeted compounds were analyzed and identified by comparing their retention times and mass spectra with those in the NIST or WILEY library.

### Network visualization

Protein-protein interactions (PPI) were searched against geneMANNIA, BAR, STRING, mentha, Interoporc, IntAct, DIP, APID, MINT, and BIND PPI databases and the *in silico* generated patho-stress responsive protein network was visualized with Cytoscape version 3.0.2 (Shannon et al., 2003). A list of 43 non-redundant proteins identified in this study was uploaded into the Cytoscape and PPI was predicted for 12 proteins.

### Statistical analysis

The statistical significance of each of the time point dataset on the normalized spot volumes were evaluated by One-way ANOVA (*p* < 0.05) with Bonferroni post-hoc correction using MeV (Saeed et al., 2003). Principal component analysis was performed using PCA functions of the XLStat Pro Version 2012.4.03 (http://www.xlstat.com) software. Values of all parameters analyzed are of three biological replicates per sample.

## Results

### Patho-stress induced changes and identification of patho-stress responsive proteins

**Figure 1:**
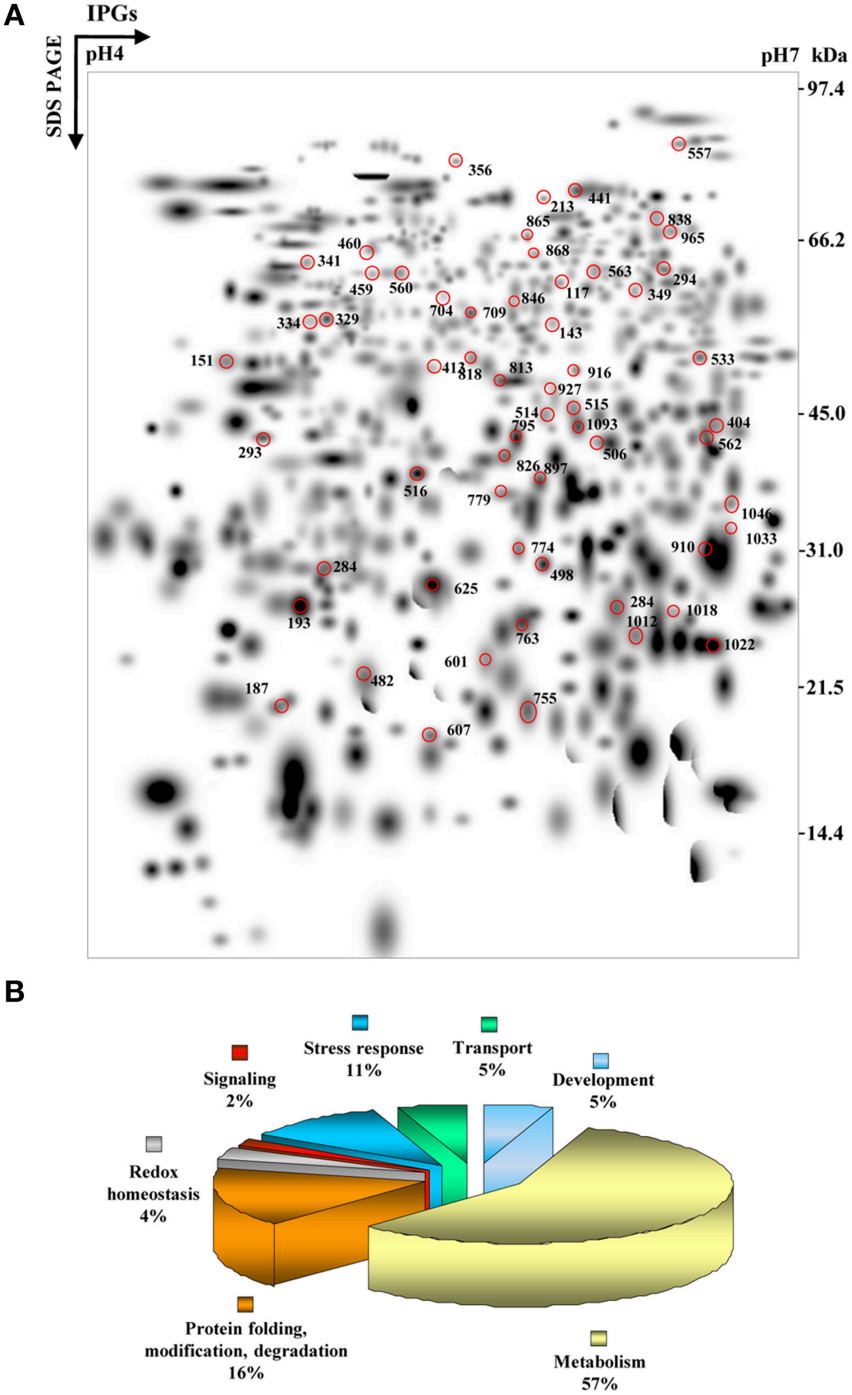
**Higher level match set and functional cataloging of PSRPs. (A)** The match set was created *in silico* from three standard gels for each of the time points as depicted in Supplementary Figure S1. The numbers indicate spots listed in Supplementary Table S2 identified by MS/MS analysis. **(B)** Pie chart showing the functionally categorized PSRPs.

To understand the necrotrophic mode of disease development associated with non-host response during storage, tomato fruits were infected with *Sclerotina rolfsii* in a time course experiment up to 120 hpi. There were no visible changes in the fruits until 24 hpi, but white mycellial mat appeared after 48 hpi and the damage was further aggravated during 96–120 hpi (Supplementary Figure S3). The temporal proteomic changes were monitored using high resolution 2-DE of total proteins from unstressed and patho-stressed fruits. We detected 382 high quality spots at 48 hpi and disease severity curve also showed increase in disease area from 24–48 hpi. There was a plateauing in the disease area from 72 to 96 hpi correlating with comparatively less number of detected spots of 278 and 287, respectively. At 120 hpi, there was again an increase in disease area paralleling higher number of detected spots as 341. A second level matchset was created that consisted of 650 total spots of which 216 showed differential abundance (log_2_ > 1.32, *p* < 0.05) at one or more time points (Figure 1A, Supplementary Table S2).

MS/MS analysis led to the identification of 56 PSRPs and 1 fungal protein with a significant match against a combined database search (Supplementary Table S2). Fifty-Six PSRPs account for 43 distinct proteins suggesting 77% unique protein identification, while the remaining 23% either correspond to post-translationally modified forms or members of multi-gene families based on sequence alignment and PTM analyses (Supplementary Table S6). Of the 56 PSRPs, 22 spots were upregulated, 28 spots showed downregulation while six exhibited mixed pattern of temporal abundance (Supplementary Table S2). The identified fungal protein showed increased abundance (Supplementary Table S2). Proteins with the respective MS/MS spectra are provided in Supplementary Table S3.

Many seemingly well-resolved 2-DE spots were found to contain more than a single protein. In an attempt to minimize co-migration of multiple protein species, we used a relatively narrow pH range (4–7) for IEF, as narrow range IPG strips facilitates higher resolution (Sghaier-Hammami et al., 2009). The top-ranked hit from LC–MS/MS analysis has been shown to correspond to the most abundant protein among multiple proteins present in a spot (Yang et al., 2007). The spot intensities of different protein constituents were determined using the emPAI (Supplementary Table S4), which have been routinely applied in proteomics workflows (Ishihama et al., 2005). Taken together, the effects of co-migrating proteins on the protein expression ratios observed in 2-DE analyses were deemed negligible. In cases where more than one protein was indicated with a significant score for the MS/MS derived peptide sequence, the match was considered in terms of the highest ranked hit, molecular mass matches, and emPAI.

### Explorative statistics reveals relevant clusters of co-regulated proteins

To describe the proteomic dataset with exploratory statistic in response to patho-stress, differentially abundant spots from total dataset were subjected to PCA to interpret proteome data for significant variability and relevance with respect to time kinetics (Supplementary Figure S4A). Furthermore, scree plot of time kinetics data from 56 PSRPs reflected variation through four principal components (Supplementary Figure S4B). Biplot analysis showed that on plotting PC1 (33.6%) against PC2 (20.07%) revealed distribution of protein spot into three clusters. The PCA analysis and the biplot revealed a time dependent patho-stress related protein abundance dynamics. Proteins that showed significant difference in abundance (marked by a circle) may be considered as potential markers in patho-stress response (Supplementary Figure S4B).

### Functional distribution and dynamics of PSRPs

To elucidate the function, 56 PSRPs were sorted into seven functional categories (Figure 1B and Supplementary Table S2). Major functional category corresponded to proteins involved in metabolism (57%), followed by protein folding, modification, and degradation (16%), next stress response (11%). These proteins represent cellular and organic substance metabolic process that relate to ion binding, small molecule binding and oxidoreductase activity (Supplementary Figure S5). The identified fungal protein belonged to the category of unknown function (Supplementary Table S2).

**Figure 2:**
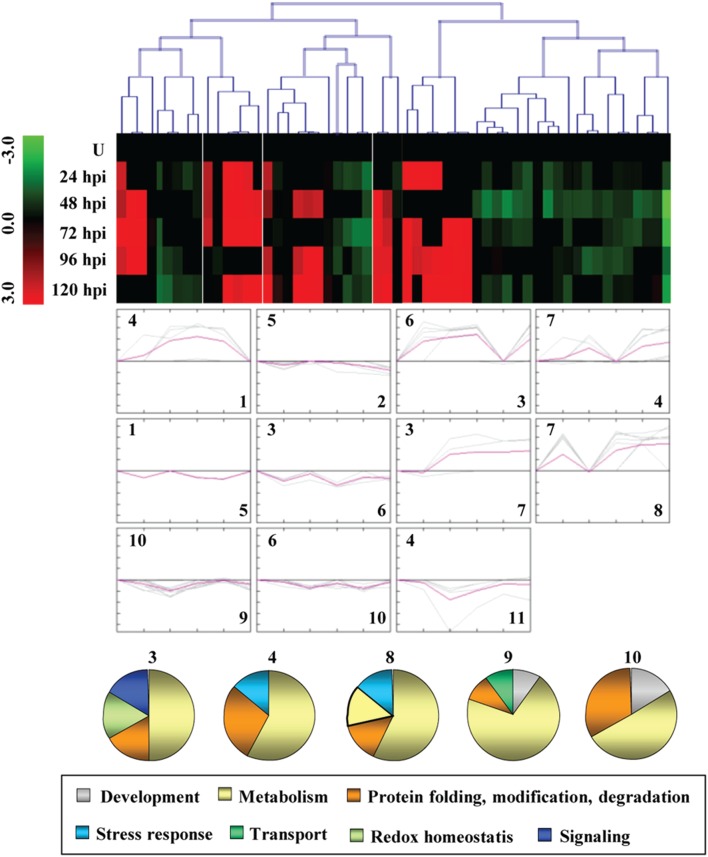
**Clusterogram of PSRPs**. Fifty-six identified PSRPs were grouped into 11 clusters based on their expression pattern. The SOTA cluster tree is shown at the top, and the expression pattern in clusters are shown below. High (or low) abundance ranges from pale to saturated red (or green). U, unstressed.

To study the correlated abundance pattern of PSRPs, SOTA clustering was performed. The analysis yielded 11 expression clusters, where clusters with *n* > 5 were taken into consideration (Figure 2, Supplementary Figure S6). The most abundant group, Cluster 9 was found to be dominated by metabolic, development, protein turnover, and transport proteins. Cluster 4 and 8 included proteins that were mixed regulated and involved in metabolism and protein turnover. While Cluster 1 and 2 consisted of proteins downregulated in later stages of infection, proteins in Cluster 3 and 8 showed upregulation during initial stages of infection. Cluster 2 was dominated by proteins which showed downregulation during pathogen invasion.

### Correlation network of PSRPs

**Figure 3:**
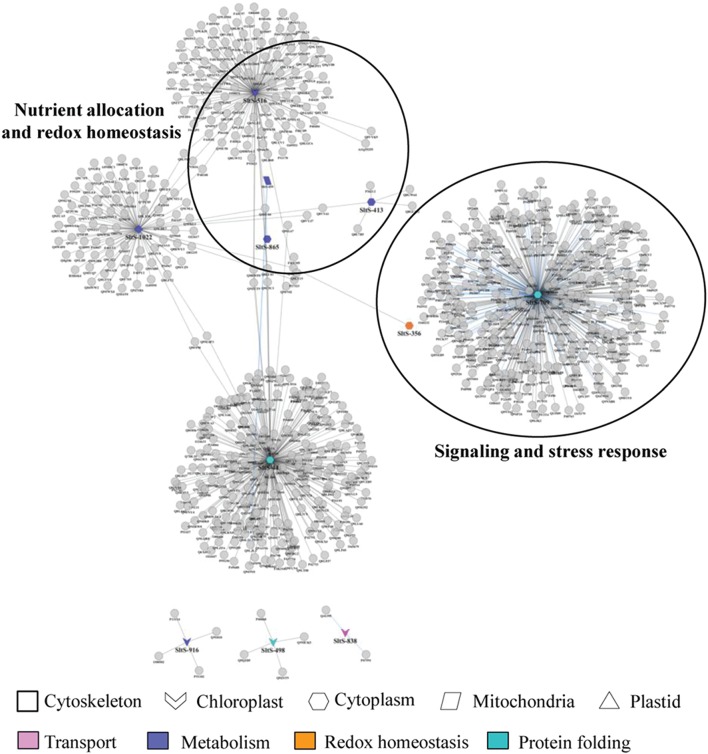
**A comprehensive protein-protein interaction network of identified PSRPs**. A spoke network of the identified PSRPs were interconnected by querying PPI databases with all identified proteins for experimentally determined interactions as listed in manually curated databases. Node colors represent functional categories while node shape represents subcellular localization of the protein species.

Cellular network could help built novel hypothesis about disease mechanism. To identify set of pathways related to patho-stress, we designed and assemble protein abundance data in correlation network segregated into functional hubs. The network consisted of 484 nodes and 375 edges and was enriched in proteins involved in signaling and stress response, nutrient allocation, and redox homeostasis and cellular architecture (Figure 3). Overall, we analyzed highly connected networks of functionally related proteins. Two major hubs were identified, of which hub 1 (signaling and stress response) mapped to 2 proteins involved in signal perception, transduction, regulation and stress response including peroxiredoxin (SltS-356), and T-Complex protein 1 subunit gamma (SltS-709). Hub 2 comprised of four candidate proteins related to nutrient allocation and redox homeostasis. The abundance patterns of malate dehydrogenase (SltS-516), 4-hydroxy-3-methyl-2-enyl-diphosphate reductase (SltS-413), fatty acid oxidation complex subunit alpha (SltS-865), and ATP synthase subunit beta (SltS-459) indicate role of primary metabolism and oxidative burst during pathogen invasion. The two co-regulated carbohydrate biosynthetic enzymes, malate dehydrogenase, and ATP synthase beta are critical to maintain the metabolic state of the patho-stressed fruit possibly by modulating TCA cycle and electron transport chain. Altogether this data demonstrate that protein signature and interaction can functionally link translational changes to disease response.

### Immunoblot analysis

**Figure 4:**
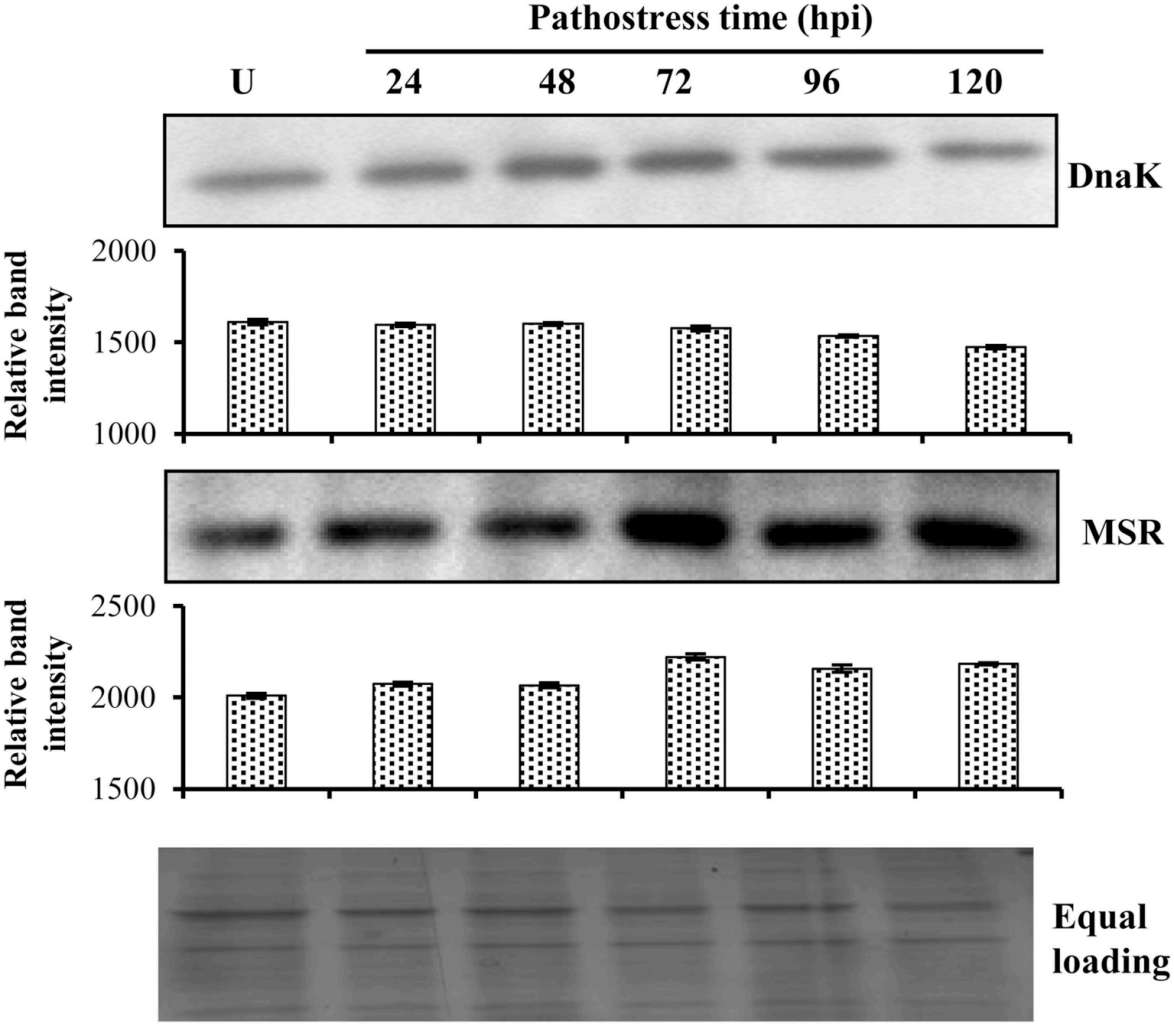
**Immunoblot analysis of selected PSRPs**. An aliquot of 50 μg soluble proteins was separated by 12.5% SDS–PAGE, blotted onto Hybond-C membrane, processed for one-dimensional immunoblot analysis and quantified by densitometry. Immunoreactive protein levels for DnaK and MSR was detected using the respective primary antibodies and signals were detected. U represents unstressed fruit. Commassie blue-stained gel indicates equal protein loading across time points.

While many of the PSRPs found in the present study have earlier reported in disease responsive proteome, some were found to be unique in our study. Of these, unique proteins those may be associated with disease response during the challenge of a necrotrophic pathogen in storage organ and found to be significant based on PCA analysis falling into the network (Supplementary Table S2) were selected for analysis by western blot. Further, chaperone DnaK and MSR migrated in multiple spots (Figure 4). The abundance of chaperone DnaK decreased with subsequent time points. The MSR more or less followed the same trend as was revealed from densitometric analysis in corroboration with the 2-DE analysis (Supplementary Table S2). Although, a similar trend in abundance was observed the difference in fold-induction may be attributed to different protein isoforms or use of polyclonal antibodies.

### Expression levels of key genes encoding PSRPs

**Figure 5:**
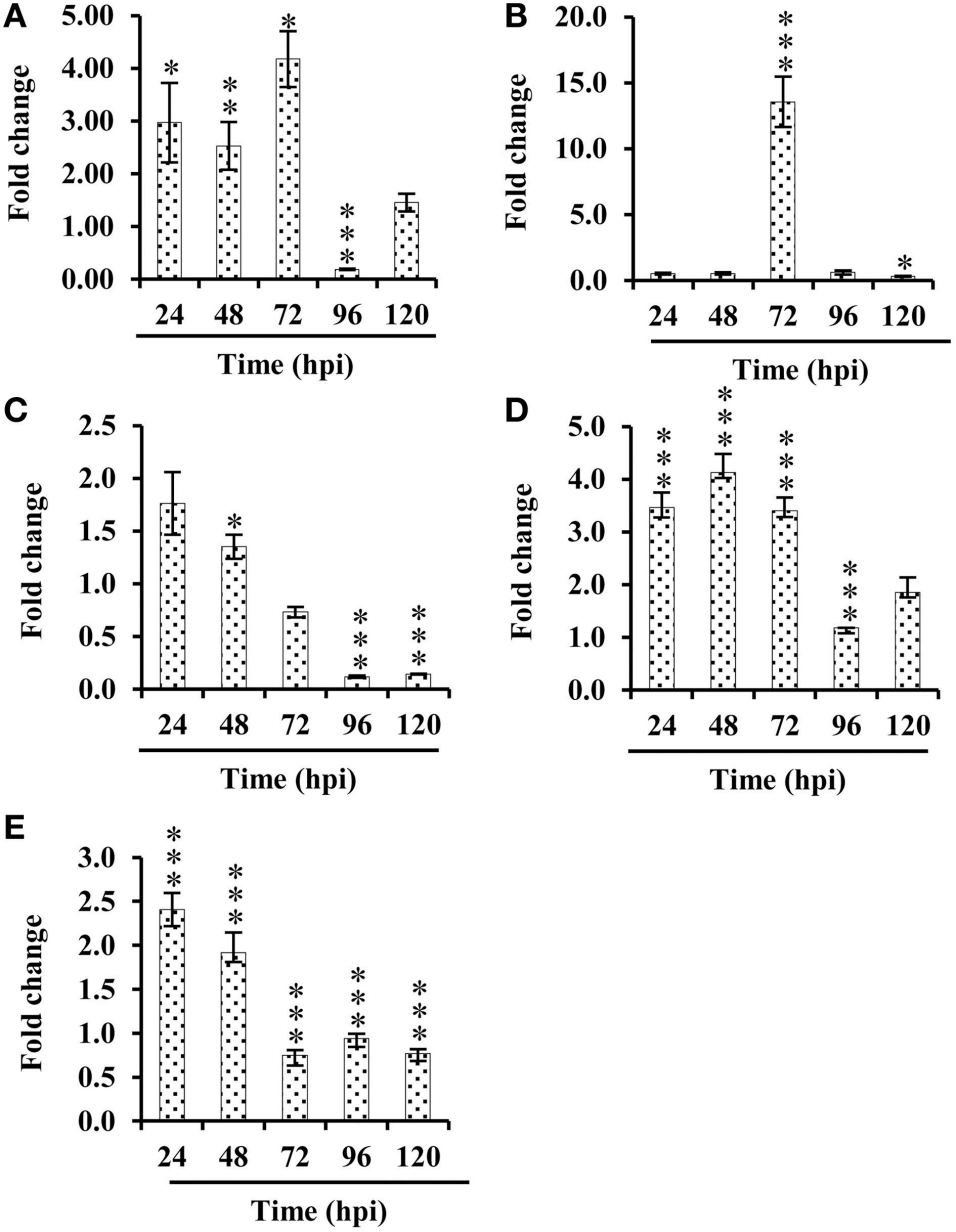
**Relative quantification (RQ) of mRNA levels**. Five candidate genes differentially expressed in tomato fruits challenged with *S. rolfsii* at 24, 48, 72, 96, 120 hpi. Expression changes were analyzed by ANOVA and vertical bars indicates SE. Significantly up- or down-regulated genes are indicated by ^*^p < 0.05 (Tukey *post-hoc* test). **(A)** 1-aminocyclopropane-1-carboxylate oxidase, **(B)** Stress induced protein sti1-like protein, **(C)** Actin depolymerizing factor, **(D)** Inositol monophosphatase 3, **(E)** late embryogenesis abundant protein.

To gain mechanistic insight of disease regulation at the mRNA level, we further investigated transcript abundance of the five selected key genes encoding PSRPs found in the network, in the patho-stressed condition of tomato fruits using the qRT-PCR (Figure 5). The expression behavior of 1-aminocyclopropane-1-carboxylate oxidase, and actin depolymerizing factor in response to *S. rolfsii* was in accordance with the proteome study, showing a similar profile with enhanced expression from 24 to 120 hpi, except 96 hpi. The transcription profile of stress induced protein sti1-like protein revealed an upregulated expression pattern from 24 to 120 hpi. However, Inositol monophosphatase 3 showed mixed expression pattern. Furthermore, actin depolymerizing factor and late embryogenesis abundant protein showed downregulation in later stages of invasion.

### Comparative analysis of differential metabolites

**Figure 6:**
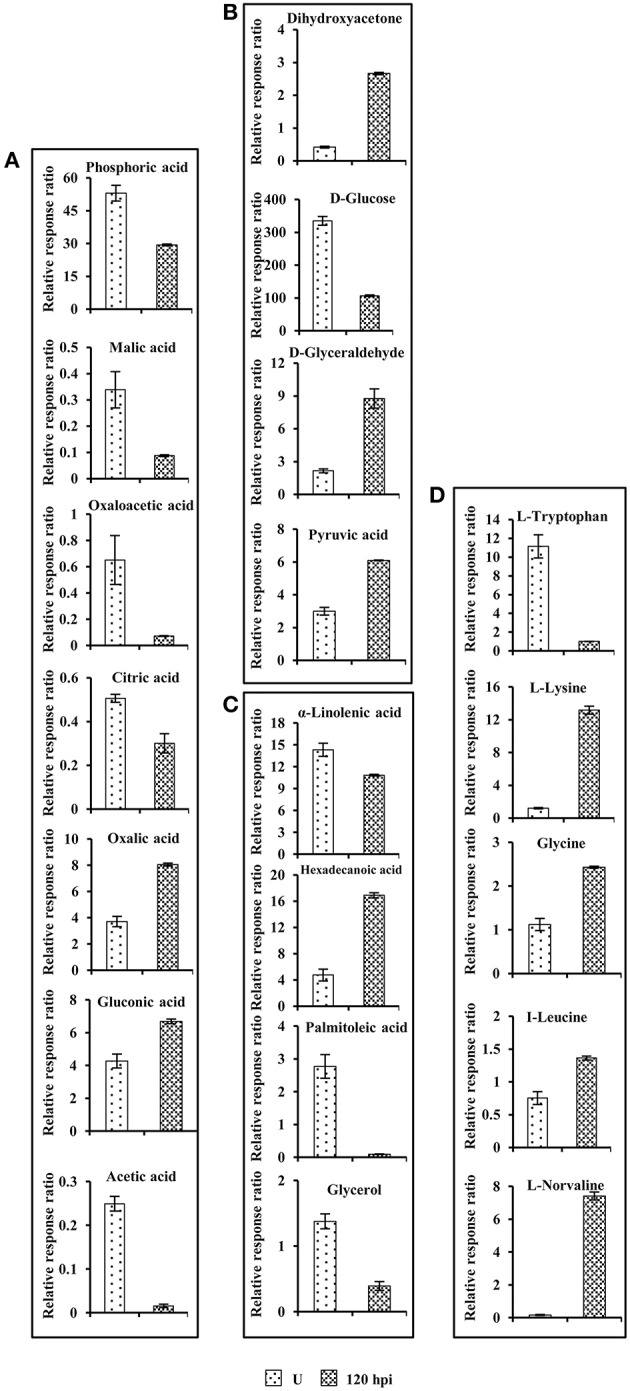
**Comparison of primary metabolite levels in unstressed tomato fruits with those in tomato fruits challenged with ***Sclerotinia rolfsii*** at 96 hpi**. **(A)** Organic acids, **(B)** Carbohydrates, **(C)** Fatty acids and, **(D)** Aminoacids. Data are normalized to the mean response calculated for unstressed levels of each replicate (to allow comparison between replicates, individual values were normalized in same way). Values presented are the mean ± SD of four independent replicates.

Next, we evaluated and analyzed the metabolite pools using GC-MS to understand the impact of PSRPs on the primary metabolism in pathogen challenged fruit. Targeted compounds were identified to corroborate with the biological pathways based on the proteomic analysis. Seventy-two metabolites were identified in unstressed fruit and 78 metabolites detected in patho-stressed (120 hpi) fruits, respectively. Of the 56 common metabolites between unstressed and patho-stressed fruits, 36 (64%) were up-regulated, whereas 20 (36%) were down-regulated. The primary metabolites related to disease pathways are shown in Figure 6 and Supplementary Table S7. Consistent with the proteomic data, there was remarkable difference in metabolite profile with high accumulation of oxalic acid, the pathogenicity determinant in stressed fruit. Metabolites related to hydrophobic amino acid biosynthesis showed high levels. Notable trend in the levels of organic acids was the decrease in the concentration of malic acid and oxaloacetic acid by a factor of 3.80 and 8.9. Furthermore, acetic acid, a short chain organic acid showed striking decrease of 13.0 times, which indicates the accumulation of glycolytic cycle metabolites. Indeed, metabolites related to carbohydrate biosynthesis were significantly altered in patho-stressed fruit. As expected, the levels of D-glucose and D-ribose were decreased in patho-stressed fruit, in accordance with the proteome data (Figure 6). However, some metabolites mostly the catabolic by-product of carbohydrates responded in opposite directions, namely dihydroxyacetone, glyceraldehyde, and pyruvic acid that showed increase in abundance during invasion.

## Discussion

**Figure 7:**
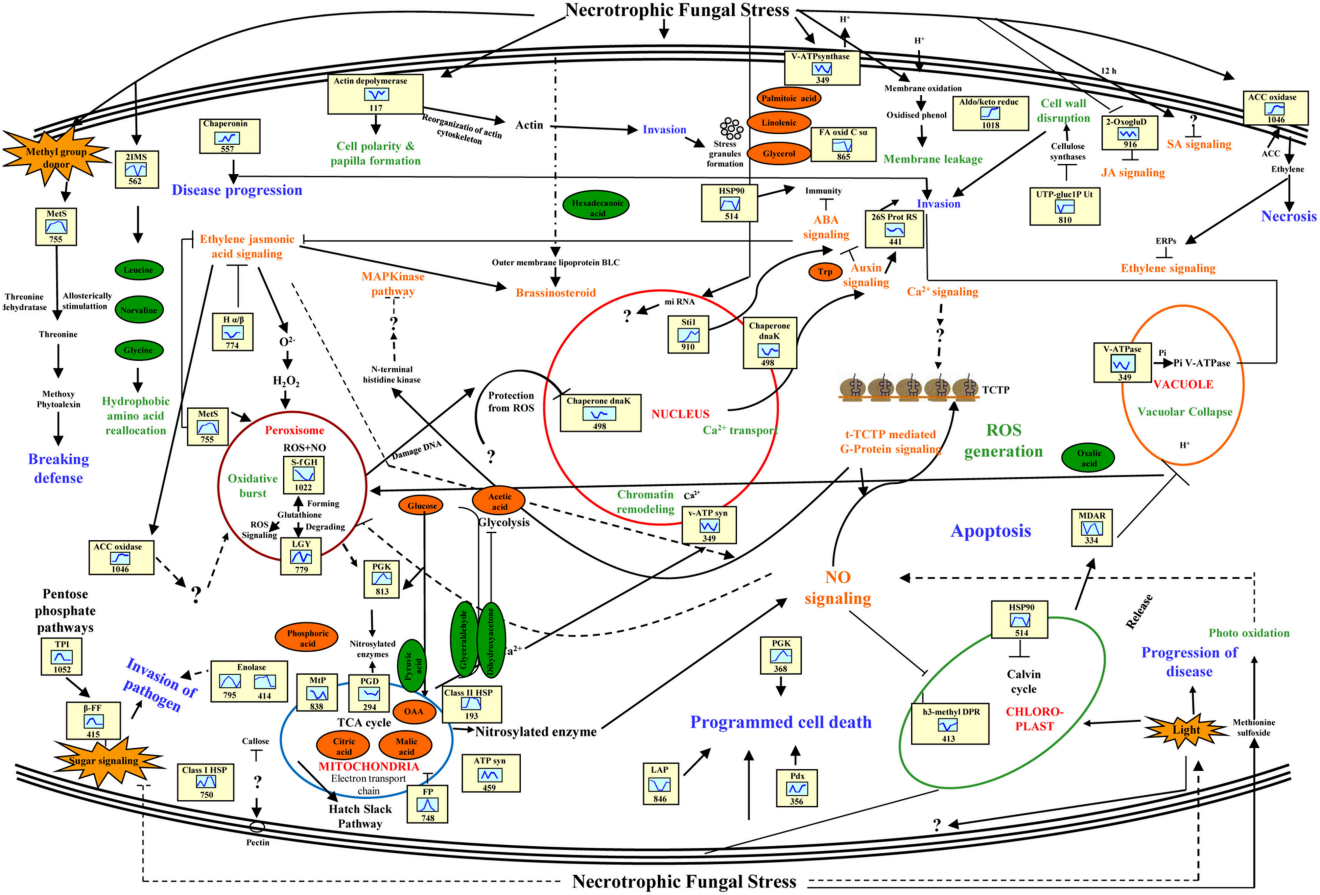
**Pathway involved in crosstalk among diverse pathways putatively functional in disease responses of Tomato-***Scleretonia*** interaction**. Proteins identified in this study are indicated in the yellow boxes. Green circles represent metabolites up-regulated, and orange circles indicate down-regulated metabolites. The graphs are representative of the abundance pattern of individual protein, and the number shown below indicates the protein identification number. LEA, late embryogenesis abundant protein; S-AHC, S- adenosylhomocysteinase; APx, PGK, phosphoglycerate kinase; PP, pyrophosphatase; NO, nitric oxide; MtP, mitochondrial peptidase; C-I HSP, C-II HSP, GST, glutathione S-transferase; ACC, ROS, reactive oxygen species; β-FF, beta-fructofuranosidase; GAPDH, MDH, IMS, 2-isopropyl malate synthase; CA, carbonic anhydrase; FP, KARI, ketole acid reductase isomaerase; PGD, phosphoglycerate dehydrogenase; PGK, Prx, LGY, lactoyl glutathione lyase; AOD, acetylornithine deacetylase; LAP, leucyl amino peptidase.

The interaction between phyto-pathogenic fungus and fruit results in rapid and highly structured multicomponent responses in both protagonists. Our study provides an overview of changes in fruit proteomes during early, intermediate, and later stages of infection by *S. rolfsii* during storage. This study also confirms the impact of patho-stress on overall cellular physiology and provides new perspectives to study the cell death response during post-harvest storage in a sink tissue. A model representing regulatory and functional networks activated under patho-stress is depicted in Figure 7.

### Nutrient metabolism and reallocation in patho-stressed fruit

Carbon and nitrogen is essential for survival of both host and pathogen and nutrient exchange between them results in disease development (Howlett, 2004; Dulermo et al., 2009). During a compatible interaction, competition of the fungus with sink organs of the host results in considerable modification of photo-assimilate production and alterations in assimilate partitioning within host tissues. A total of 32 identified PSRPs in infected tomato fruit were found to relate to metabolism, including carbohydrate (22), amino acid (6), and fatty acid metabolism (4). Concerted action of these PSRPs might contribute to metabolic reprogramming in the tomato fruit during fungal invasion.

Repression of photosynthesis is a general response of host-pathogen interaction (Jobic et al., 2007). Our study revealed downregulation of 4-hydroxy-3-methylbut-2-enyl diphosphate reductase (SltS-413; –1.44 to –0.47-fold) and carbonic anhydrase (SltS-563; –0.05 to –0.08-fold) in later and early stage of invasion, respectively while phosphoglycerate kinase (SltS-813; –0.27 to 0.53-fold) showed mixed abundance. Pathogenesis also involves conversion of sugar to sugar alcohols in fruit (Dulermo et al., 2009). We observed upregulation of aldo-keto reductase (SltS-1018; 3.3–3.7-fold) at 72 and 96 hpi, which might increase polyol pool during pathogenic interaction. Indeed, metabolite profiling revealed increase in mannitol (polyol) in patho-stressed fruit. Furthermore, SltS-413 and SltS-1018 were grouped in same cluster in PCA analysis showing significant relevance and variation in diseased state. However, these proteins appeared in two different clusters of dendrogram namely cluster 3 and 8 based on their differential abundance pattern. Besides, sugar phosphates suppress cellulose synthesis by feedback mechanism preventing fortification of the cell wall. It is clear that there are multiple levels at which regulation of cellulose synthesis occurs (Kleczkowski et al., 2011). Our result indicated that altered regulation of cellulose biosynthesis enzyme viz., hydrolase alpha/beta fold family protein (SltS-774; –1.1 to –0.15-fold) leading to disease progression.

Fermentative catabolism in fruits plays an important role during plant–pathogen interactions (Dulermo et al., 2009). Our analysis revealed that acid beta-fructofuranosidase (SltS-329; 2.25–2.86-fold), and enolase (SltS-795 and 818; 2.02–3.5-fold) were upregulated during pathogenesis. It was interesting to note increased accumulation of glycolytic pathway associated metabolites like glyceraldehyde, dihydroxyacetone and pyruvic acid, while glucose showed decrease in abundance. Pan et al. (2013) previously had reported upregulation of enolase in tomato-*Rhizopus* interaction. Therefore, silencing enolase might be a strategy to combat fungal disease. The results also showed that proteins involved in aerobic respiration like malate dehydrogenase (SltS-1093; –1.9 to –0.6-fold) was downregulated possibly due to its diversion to C3 and amino-acid metabolism. Likewise, decreased levels of malic acid, citric acid, and oxaloacetic acid confirmed alterations in sugar metabolism-related expression implying therby carbon metabolism imbalance during patho-stress. Earlier virus induced gene silencing of malate dehydrogenase in a resistant cultivar of tomato was shown to make the crop susceptible to the powdery mildew fungus, *Oidium neolycopersici* (Pei et al., 2010).

Free amino acids also represent an important sink of absorbed and assimilated carbon for fungal invasion (O'Donnell et al., 2003). Further, plant-fungus interaction can enhance expression of genes involved in amino acid recycling, proteolysis, and transport (Huber and Watson, 1974). We found that enzymes related to amino acid metabolism were affected due to *S. rolfsii* invasion that might further methoxylate phytoalexins and break host defense by providing nutrient source for the invading pathogen. Strikingly, hydrophobic aliphatic amino-acid biosynthesis enzymes, viz. 2-isopropylmalate synthase 1 (SltS-562; –0.12 to 0.3-fold) of leucine, valine, and glycine biosynthesis showed differential regulation. The metabolite profiling of hydrophobic amino acid complement showed increased levels of glycine, leucine, norvaline, and norleucine that confirmed diversion of glycolytic metabolites to hydrophobic aminoacid biogenesis. The amino acid reallocation due to fungal attack might affect metabolism of basic and S-containing amino-acid. Methionine sulfoxide reductase (SltS-755; 2.7-fold), and acetylornithine deacetylase (SltS-341; 2–3.4-fold) were upregulated possibly distributing stored nitrogen to modulate plant metabolism. The increased level of basic amino acid namely lysine during patho-stress regulate disease state during invasion. Additionally, the expression behavior of tomato methionine sulfoxide reductase in response to *Sclerotinia* infection was in complete accordance with the proteome analysis, showing a very similar profile with enhanced abundance (Figure 4).

Another protein, fat biosynthetic enzymes like fatty acid oxidation complex subunit alpha (SltS-865; –1.1 to –0.06-fold) was downregulated suggesting that disease progression possibly occur by detoxifying lipid and curbing lipid hormone synthesis in later stages of invasion. Metabolite profiling also revealed that alpha linolenic acid, palmitoleic acid, and glycerol had decreased level during patho-stress. The potentiated responses include oxidative burst, signal transduction, protein turnover, transport, and developmental changes.

### Signal transduction networks in patho-stressed tomato

Metabolic control and responses to fungal invasion are function of complex signal transduction pathways. *Sclerotinia* is known to regulate production and signaling responses of plant hormones during plant-pathogen interaction (AbuQamar et al., 2006). In the present study, there were nine identified PSRPs involved in signal transduction, including ligand binding to receptors, generation of second messengers, and related cascades.

It has been suggested that translational changes in fungal invaded fruit show complementary action of jasmonic acid (JA) and ethylene (ET) promoting susceptibility (AbuQamar et al., 2006). Consistent with the earlier findings, 14-3-3 (SltS-704; –4.6 to 0.8-fold), 2-oxoglutarate dioxygenase (SltS-404 and 916; –1.04 to –0.01-fold), and late-embryogenesis abundant protein 2 (SltS-284; –0.69 to –0.64-fold) were differentially abundant indicating synergistic interaction between JA and ET. The q-RT PCR analysis revealed that expression of late-embryogenesis abundant protein 2 (Figure 5E) was significantly downregulated in patho-stressed fruit during later stages of invasion. Furthermore, deregulation of ACC oxidase-like proteins (SltS-826, 1033 and 1046; –0.96 to 3.3-fold) might be due to combinatorial sensing and inhibition of ET along with JA causing senescence and necrosis. Fungal invasion resulted in the increased transcription of ACC oxidase, with a maximum level of gene expression (3.00-fold) at 72 hpi (Figure 5A).

It is implicated that imbalances in auxin mediated signal cascades suppress proteosome machinery to promote protein stability (Howe and Schilmiller, 2002). 26S protease regulatory subunit 6A homolog (SltS-441; –0.5 to –0.26-fold) showed down-regulation at initial stage, indicating inhibition of auxin related signaling due to necrotroph invasion. Corroborating this finding, metabolite analysis also revealed decrease levels of tryptophan and acetic acid, the precursors of auxin biosynthesis.

### Redo homeostasis and protein modification regulate the spread of disease in tomato fruit

Plant response to fungal invasion includes ROS production, oxidative damage, and hyperosmotic stress. A total of 7 PSRPs were involved in redox homeostasis, whereas 16 were related to protein folding, modification and degradation. ROS are toxic byproducts of aerobic metabolism and an indicator of stress-induced damage. Other auxiliary elements (molecular chaperones) also participate in protein refolding under stress. Molecular chaperones, HSP family class II HSP (SltS-193; 2.82–3.1-fold), and chaperone DnaK (SltS-560; 0.006–0.02-fold) are regulatory centers for plant biological processes. Notably, qRT-PCR analysis showed that chaperone DnaK exhibited steady state level till 72 hpi followed by gradual decrease (Figure 4). Our study underscores that mitochondrial processing peptidase alpha subunit (SltS-838; –0.85 to –0.1-fold) was differentially expressed to modulate feedback fluxes and protein homeostasis in the host.

ROS also regulate signaling and accumulation of chaperone under patho-stress and might fine-tune specific cellular responses. It has been reported that glutathione in ascorbate-glutathione cycle removes cytotoxic compounds (Llorentea et al., 2008). Glutathione forming enzymes, namely S-formyl glutathione hydrolase (SltS-1022; –0.6-fold) was downregulated, whereas glutathione degrading enzyme lactoylglutathione lyase (SltS-779; 2.6–4.0-fold) was upregulated. Moreover, one of the ascorbate-glutathione cycle enzymes, monodehydroascorbate reductase (SltS-334; –1.1 to –0.5-fold) showed downregulation. Metabolite analysis also showed decrease concentration of ascorbic acid in patho-stressed fruit in comparison to the control. These data is consistent with the earlier reports that glutathione signaling is inhibited during *Sclerotinia* invasion causing oxidative burst and toxification of the host cell. Additionally, necrotroph restrain ROS mediated host signaling. The critical balance between ROS signaling and cell death determine pathogenicity and disease establishment. The cascade of ROS regulated enzymes, such as, peroxiredoxin (SltS-356; 2.8-fold) showed upregulation at later stage of invasion that might facilitate activation of ROS signaling to circumvent pathogen attack. It is likely that *Sclerotinia* first establish infection due to accumulation of ROS, and then trigger rapid spread of cell death in the host.

### Cell architecture dynamics during pathogen invasion

The plant cytoskeleton sense and respond to phytopathogen by expending an enormous amount of energy. Fungal stimuli cause massive depolymerization of cytoskeleton organization especially actin which is a signaling target for stress (de Torres-Zabala et al., 2007). Upregulation of Inositol monophosphatase 3 (SltS-515; 2.6–3.2-fold) and downregulation of actin-depolymerizing factor, ADF (SltS-117; –0.8 to –0.1-fold) might change cell polarity during fungal intrusion thus supporting stochastic cytoskeleton dynamics in the patho-stressed fruit. At transcript level Inositol monophosphatase 3 showed maximum increase at 48 hpi (1.12-fold) (Figure 5D). Notably, qRT-PCR analysis of actin-depolymerizing factor revealed that both transcription and protein profile were similar and exhibited gradual decrease in expression from 24–120 hpi (Figure 5C). ADF plays key role in the activation of gene-for-gene resistance during *Arabidopsis thaliana-Pseudomonas syringae* interaction (Tian et al., 2009), highlighting its importance toward conferring tolerance by over-expression in case of *Sclerotinia* rot disease.

In our study, NADP and ATP generating enzyme such as ATP synthase beta (SltS-459; 2.32–3.0-fold) was upregulated indicating energy supply for cytoskeleton reorganization during invasion. Further, actin complex formation might lead to cell death in patho-stressed fruit (de Torres-Zabala et al., 2007). A major latex like protein (SltS-460; 2.32–2.9-fold) was upregulated, but its role in patho-stress is unknown. It is likely that this might be a necrotroph regulated protein having role in disease progression.

### Comparison of patho-stress responsive proteomes

**Figure 8:**
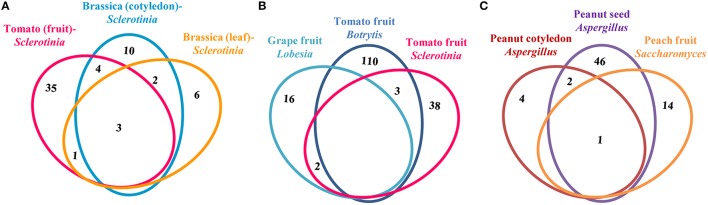
**Comparative analyses of patho-stress proteomes**. Venn diagram showing overlapping proteins during **(A)** *Sclerotinia* interaction with host storage organs, **(B)** Compatible interaction of necrotrophs with host storage organs, and **(C)** Incompatible interaction of necrotrophic fungi with host storage organs. The numbers signify the unique and/or orthologous proteins among the organisms studied. The areas are not proportional to the number of proteins in the groups.

To understand general vs. specific host response in necrotrophic pathogenesis, we compared compatible and incompatible interaction proteome datasets at different levels based on the pathogen in question and diverse host. First, we studied *Sclerotinia* responsive proteomes during compatible interaction involving different host and diverse organs reported till date (Figure 8A). Analysis of tomato (fruit)-*Sclerotinia* (this study), *Brassica* (cotyledon)-*Sclerotinia* (Garg et al., 2013), and *Brassica* (Leaf)-*Sclerotinia* (Liang et al., 2008) revealed three common proteins of glycolytic pathway and TCA cycle associated with carbohydrate metabolism. Hexokinase, triose phosphate isomerase, and malate dehydrogenase showed similar function in different host interactions with *Sclerotinia*. It was interesting to note that specificity in functionality was in the protein degradation machinery of the host. While *Sclerotinia* invaded tomato fruit seems to utilize proteosome mediated degradation of misfolded proteins, proteomes of *Sclerotina* invaded *Brassica* leaves and cotyledons showed predominance of peptidases and protein inhibitors, respectively to remove partially degraded proteins from the tissue. Therefore, it appears specific response of different proteins culminates in the same function in different host invaded with the same pathogen.

Secondly, to determine commonality and diversity among differentially abundant proteins in storage organs during compatible interaction with necrotrophic colonization, we compared tomato (fruit)-*Botrytis cinerea* (186 proteins) and grape (fruit)-*Lobesia botrana* (20 proteins) with that of tomato (fruit) infected with *S. rolfsii* (43 proteins) proteomes (Figure 8B). It was observed that two proteins belonging to fatty acid metabolism namely, desaturase and lipoprotein oxidase represent the social class. The analysis further depicts that proteins belonging to metabolism (32%) contribute majorly to each of these proteomes. Diversity of proteins involved in cAMP signaling and redox homeostasis was reported in tomato–*Botrytis* proteome (Shah et al., 2012). However, G-protein signaling components, such as translationally controlled tumor protein was differentially abundant in *Sclerotinia* infected tomato fruit. No protein related to such signal transduction was reported in grape (fruit)-*Lobesia botran* (Melo-Braga et al., 2012). This suggests, although the nature of the integral protein candidates in different signaling pathway differs, but they show similar expression dynamics ultimately culminating to the compatible interaction to have the same effect on the host phenotype.

Thirdly, to elucidate the general and specific response of incompatible interactions in storage organ, we compared peanut (seed)-*Aspergillus flavus* (Wang et al., 2010), peanut (cotyledon)-*Aspergillus flavus* (Wang et al., 2012), and peach (fruit)-*Pichia membranefaciens* (Chan et al., 2007) proteomes (Figure 8C). One well-known cytoskeleton protein, namely, actin was found to be common in these three pathosystems, suggesting its general function toward maintenance of mechanical property. Peanut (seed)-*Aspergillus flavus* and *peanut* (cotyledon)-*Aspergillus flavus* proteomes had two common proteins belonging to primary metabolism, including 2-oxoglutarate dioxygenase and aldolase. However, specificity in function is observed in all three system related to proteins involved in redox homeostasis and cell wall architecture regulation during incompatible interactions.

Overall, the comparative analyses revealed multi-specificity in protein degradation, redox homeostasis and cell wall architecture dynamics, while metabolism is a general response in both compatible and incompatible interaction.

## Conclusion

In summary, we explored tightly regulated and interconnected processes combining multifactorial time series analysis with information extracted from system level data describing molecular disease phenotype in necrotrophic fungal invasion during post-harvest storage. Integrative comparative analysis of proteome from patho-stressed tomato fruits identified common and distinct molecular events and unique correlations during disease progression. Canonical proteins like enolase, malate dehydrogenase, and ADF involved in plant-pathogen interaction are interesting candidates for future investigations. Novel findings include discovery of major latex protein whose role in necrotroph invasion is not known and can be used as biomarker. These potential proteins may be taken for crop improvement program either manipulated by over-expression or downregulation. Indeed, our future efforts will be focused onto characterizing some of these potential candidates. Taken together, these data greatly improve our understanding of disease progression and cell death as well as provide the basis for hypothesis-driven research for targeted alteration of metabolic routes for effective engineering strategies to combat plant disease and restoration of post-harvest quality.

## Author contributions

SC conceived the study. SC, SG, RG, KN designed the study. SG, KN, AS, PJ, and RG performed the wet-lab experiments. SG, KN, and AS performed proteomic analysis. SC, SG, KN, NC, and SC contributed to data analysis and interpretation. SC, SG, and KN wrote the manuscript.

### Conflict of interest statement

The authors declare that the research was conducted in the absence of any commercial or financial relationships that could be construed as a potential conflict of interest.
