# Supplementary material for: Proteometabolomic Study of Compatible Interaction in Tomato Fruit Challenged with Sclerotinia rolfsii Illustrates Novel Protein Network during Disease Progression
Source: Front Plant Sci. 2016 Jul 26;7:1034. doi: 10.3389/fpls.2016.01034 (PMC4960257; doi:10.3389/fpls.2016.01034)
Supplement: Supplementary file 2 [file Table2.DOCX]

**Supplementary Table S2.** List of patho-stress responsive proteins (PSRPs) identified by MS/MS analysis.

| Functional Category | Cellular compartment^a^ | Spot ID^b^. | SOL ID^c^ | Protein Name | Time Kinetics (hpi)  U 24 48 72 96 120 | Score | NP^d^ | cover-age | Thr. MW/PI | Exp. MW/PI |
| --- | --- | --- | --- | --- | --- | --- | --- | --- | --- | --- |
| Development | Cytoplasm | *SltS-117 | Solyc06g005360.2.1 | Actin depolymerizing factor |  | 226 | 3 | 32 | 16.065/6.15 | 62.2346/5.7238 |
|  | Cytoplasm | SltS-284 | Solyc08g015690.2.1 | Late-embryogenesis abundant protein 2 |  | 141 | 32 | 21 | 35.226/4.66 | 30.7493/6.4089 |
|  | Cytoplasm | *SltS-143 | Solyc08g015690.2.1 | Late-embryogenesis abundant protein 2 |  | 63 | 3 | 10 | 35.226/4.66 | 50.8285/5.5135 |
| Metabolism | Cytoplasm | SltS-1033 | Solyc07g049530.2.1 | 1-aminocyclopropane-1-carboxylate oxidase |  | 250 | 33 | 23 | 36.018/5.13 | 34.2432/6.9427 |
|  | Cytoplasm | SltS-1046 | Solyc07g049530.2.1 | 1-aminocyclopropane-1-carboxylate oxidase |  | 600 | 48 | 40 | 36.018/5.13 | 36.9356/6.3621 |
|  | Cytoplasm | SltS-826 | Solyc09g089580.2.1 | 1-aminocyclopropane-1-carboxylate oxidase-like protein |  | 585 | 42 | 40 | 41.507/5.61 | 41.3685/5.1869 |
|  | Chloroplast | SltS-562 | Solyc08g014130.2.1 | 2-isopropylmalate synthase 1 |  | 1008 | 60 | 36 | 67.526/5.81 | 43.5301/6.5357 |
|  | Cytoplasm | *SltS-916 | Solyc02g062500.2.1 | 2 oxoglutarate dioxygenase |  | 138 | 3 | 9 | 36.734/5.17 | 50.116/ 5.9669 |
|  | Cytoplasm | *SltS-404 | Solyc02g062500.2.1 | Oxoglutarate dioxygenase |  | 163 | 3 | 9 | 36.73/ 5.17 | 46.9725/6.4736 |
|  | Cytoplasm | *SltS-533 | Solyc12g005860.1.1 | 3 isopropylmalate dehydratase large subunit |  | 296 | 12 | 14 | 107.83/6.52 | 49.7196/6.3831 |
|  | Cytoplasm | SltS-413 | Solyc01g109300.2.1 | 4-hydroxy-3-methylbut-2-enyl diphosphate reductase |  | 549 | 51 | 32 | 52.147/5.38 | 55.1654/5.0447 |
|  | Cytoplasm | SltS-341 | Solyc08g076970.2.1 | Acetylornithine deacetylase or succinyl-diaminopimelate desuccinylase |  | 302 | 39 | 30 | 43.329/5.00 | 84.146/ 4.7683 |
|  | Vacoule | SltS-329 | Solyc03g083910.2.1 | Acid beta-fructofuranosidase |  | 66 | 16 | 6 | 71.602/5.54 | 71.4293/4.8767 |
|  | Chloroplast | SltS-763 | Solyc06g065270.2.1 | Adenylate kinase |  | 144 | 17 | 21 | 32.293/6.97 | 32.9903/5.8124 |
|  | Cytoplasm | *SltS-927 | Solyc06g059740.2.1 | Alcohol dehydrogenase 2 |  | 123 | 7 | 22 | 42.582/5.97 | 69.3549/5.7663 |
|  | Chloroplast | SltS-482 | Solyc06g059740.2.1 | Alcohol dehydrogenase 2 |  | 122 | 5 | 5 | 42.582/5.97 | 86.3763/5.2508 |
|  | Cytoplasm | SltS-601 | Solyc06g059740.2.1 | Alcohol dehydrogenase 2 |  | 190 | 19 | 17 | 42.582/5.97 | 91.8401/5.394 |
|  | Cytoplasm | SltS-1018 | Solyc09g082720.2.1 | Aldo/keto reductase family protein |  | 104 | 26 | 25 | 38.682/5.91 | 20.081/ 5.0305 |
|  | Cytoplasm | *SltS-151 | Solyc09g082720.2.1 | Aldo/keto reductase family protein |  | 69 | 3 | 9 | 38.682/5.91 | 76.2475/6.3504 |
|  | Mitochondria | SltS-459 | Solyc05g008460.2.1 | ATP synthase subunit beta |  | 758 | 34 | 30 | 59.684/5.74 | 89.0524/5.0312 |
|  | Cytoplasm | *SltS-563 | Solyc05g005490.2.1 | Carbonic anhydrase |  | 43 | 2 | 8 | 29.904/5.63 | 75.0076/5.225 |
|  | Cytoplasm | SltS-795 | Solyc09g009020.2.1 | Enolase |  | 812 | 38 | 36 | 48.054/5.68 | 54.764/ 5.7857 |
|  | Cytoplasm | SltS-818 | Solyc09g009020.2.1 | Enolase |  | 1382 | 73 | 57 | 48.054/5.68 | 88.6573/5.3964 |
|  | Cytoplasm | SltS-865 | Solyc08g068390.2.1 | Fatty acid oxidation complex subunit alpha |  | 226 | 30 | 17 | 77.911/5.68 | 24.3032/6.8806 |
|  | Cytoplasm | SltS-293 | Solyc11g011380.1.1 | Glutamine synthetase |  | 126 | 5 | 6 | 38.774/5.49 | 41.3108/4.9874 |
|  | Cytoplasm | SltS-1012 | Solyc05g014470.2.1 | Glyceraldehyde 3-phosphate dehydrogenase |  | 117 | 38 | 28 | 36.822/6.34 | 63.8824/6.3688 |
|  | Cytoplasm | SltS-774 | Solyc02g065240.2.1 | Hydrolase alpha/beta fold family protein |  | 292 | 12 | 24 | 29.807/5.73 | 25.7181/6.7407 |
|  | Cytoplasm | SltS-779 | Solyc02g080630.2.1 | Lactoylglutathione lyase |  | 705 | 50 | 36 | 38.746/6.62 | 40.4713/4.3967 |
|  | Chloroplast | SltS-846 | Solyc12g010040.1.1 | Leucyl aminopeptidase |  | 946 | 35 | 33 | 60.813/7.92 | 69.402/ 5.9059 |
|  | Chloroplast | SltS-516 | Solyc03g115990.1.1 | Malate dehydrogenase |  | 203 | 21 | 18 | 43.563/8.34 | 38.3626/4.949 |
|  | Chloroplast | *SltS-897 | Solyc09g090140.2.1 | Malate dehydrogenase |  | 114 | 3 | 15 | 35.703/5.91 | 70.2191/4.9593 |
|  | Chloroplast | *SltS-1093 | Solyc09g090140.2.1 | Malate dehydrogenase |  | 159 | 3 | 15 | 35.703/5.91 | 39.5344/5.7402 |
|  | Cytoplasm | *SltS-334 | Solyc09g009390.2.1 | Monodehydroascorbate reductase |  | 169 | 4 | 9 | 47.106/5.77 | 64.9948/4.8701 |
|  | Cytoplasm | SltS-813 | Solyc07g066610.2.1 | Phosphoglycerate kinase |  | 520 | 45 | 38 | 50.592/7.66 | 78.1838/6.0379 |
|  | Vacoule | *SltS-1022 | Solyc04g016360.2.1 | S formyl glutathione hydrolase |  | 183 | 3 | 14 | 32.354/5.85 | 52.1899/6.2034 |
| Protein folding, modification, degradation | Cytoplasm | *SltS-441 | Solyc06g083620.2.1 | 26S protease regulatory subunit 4 |  | 72 | 6 | 13 | 49.566/5.91 | 75.2709/5.0905 |
|  | Chloroplast | SltS-498 | Solyc01g103450.2.1 | Chaperone DnaK |  | 1949 | 174 | 44 | 74.965/5.20 | 32.861/ 5.1529 |
|  | Chloroplast | SltS-560 | Solyc11g020040.1.1 | Chaperone DnaK |  | 1134 | 102 | 39 | 74.619/5.36 | 81.8174/5.3882 |
|  | Chloroplast | SltS-557 | Solyc01g028810.2.1 | Chaperonin |  | 559 | 34 | 26 | 63.238/5.72 | 104.109/6.3606 |
|  | Mitochondria | SltS-213 | Solyc09g091180.2.1 | Chaperonin |  | 1067 | 88 | 49 | 61.807/5.51 | 74.6484/4.9068 |
|  | Chloroplast | SltS-625 | Solyc07g042250.2.1 | chaperonin |  | 47 | 2 | 4 | 26.603/6.85 | 81.7255/5.1934 |
|  | Cytoplasm | *SltS-193 | Solyc08g062340.2.1 | CLASS II heat shock protein |  | 109 | 2 | 12 | 17.381/ 6.75 | 30.969/ 4.7372 |
|  | Cytoplasm | SltS-514 | Solyc06g036290.2.1 | Heat shock protein 90 |  | 869 | 65 | 31 | 70.444/5.20 | 44.8368/5.2112 |
|  | Cytoplasm | SltS-709 | Solyc05g056310.2.1 | T-complex protein 1 subunit gamma |  | 371 | 23 | 20 | 61.346/5.71 | 23.5121/4.6719 |
| Redox homeostasis | Chloroplast | *SltS-187 | Solyc06g060260.2.1 | Ascorbate peroxidase |  | 224 | 7 | 17 | 38.041/8.48 | 30.4187/6.713 |
|  | Cytoplasm | SltS-356 | Solyc07g020860.2.1 | Peroxiredoxin |  | 146 | 25 | 40 | 17.540/5.18 | 95.9028/4.7524 |
| Signaling | Cytoplasm | SltS-515 | Solyc11g012410.1.1 | Inositol monophosphatase 3 |  | 80 | 3 | 4 | 29.435/5.35 | 45.5106/5.358 |
| Stress Response | Cytoplasm | SltS-607 | Solyc09g090980.2.1 | Major allergen Mal d 1 |  | 234 | 28 | 64 | 17.472/5.44 | 53.0381/5.2805 |
|  | Cytoplasm | *SltS-506 | Solyc09g091000.2.1 | Major allergen Mal d2 |  | 145 | 3 | 25 | 17.456/5.67 | 43.4396/5.3155 |
|  | Cytoplasm | *SltS-460 | Solyc04g007770.2.1 | Major latex protein |  | 64 | 2 | 10 | 16.756/5.96 | 37.6929/5.3161 |
|  | Nucleus | SltS-755 | Solyc03g111720.2.1 | Peptide methionine sulfoxide reductase msrA |  | 554 | 40 | 35 | 22.247/6.10 | 53.8968/4.6028 |
|  | Nucleus | *SltS-910 | Solyc08g079170.2.1 | Stress induced protein sti1-like protein |  | 159 | 3 | 7 | 65.410/5.99 | 31.6684/6.8628 |
|  | Peroxisome | SltS-868 | Solyc08g079170.2.1 | Stress-induced protein sti1-like protein |  | 817 | 54 | 37 | 65.410/5.99 | 27.5515/6.625 |
| Transport | Cytoplasm | *SltS-704 | Solyc04g076060.2.1 | 14-3-3 |  | 63 | 5 | 25 | 28.778/4.80 | 16.4115/6.3636 |
|  | Chloroplast | SltS-838 | Solyc12g008630.1.1 | Mitochondrial processing peptidase alpha subunit |  | 586 | 31 | 23 | 54.869/6.05 | 73.7749/6.1633 |
|  | Cytoplasm | SltS-349 | Solyc12g055800.1.1 | V-type ATP synthase alpha chain |  | 419 | 36 | 21 | 68.798/5.20 | 59.0331/6.1163 |

^a^ Subcellular localization was predicted by WoLF PSORT (www.genscript.com/psort/wolf_psort.html). ^b^Spot number as given on the 2-D gel images. The first letters (Sl) signify the source plant, *Solanum lycopersicum*, followed by total protein, (t) and stressed fruit, (S). ^c^SOL Genomics Network ID. ^d^ NP represents the number of peptides. ‘*’ indicates spot identified by MALDI-TOF-TOF.

List of fungal protein identified by MS/MS analysis.

| Functional Category | Spot ID | Database ID^a^ | Protein Name | Time Kinetics (hpi)  0 24 48 72 96 120 | Score | NP^b^ | coverage | Thr. MW/PI | Exp. MW/PI |
| --- | --- | --- | --- | --- | --- | --- | --- | --- | --- |
| Unknown | 722 | SS1G_10061T0 | Sclerotinia sclerotiorum hypothetical protein |  | 63 | 7 | 53 | 125.10/7.88 | 100.53/5.66 |

^a^*Sclerotinia sclerotiorum* database ID. ^b^NP represents the number of peptides.
