# Supplementary material for: Proteometabolomic Study of Compatible Interaction in Tomato Fruit Challenged with Sclerotinia rolfsii Illustrates Novel Protein Network during Disease Progression
Source: Front Plant Sci. 2016 Jul 26;7:1034. doi: 10.3389/fpls.2016.01034 (PMC4960257; doi:10.3389/fpls.2016.01034)
Supplement: Supplementary file 5 [file Table5.DOCX]

**Supplementary Table S5.** Genes and primers used for qRT-PCR analysis.

| **Spot No.** | **Gene description** | **Accession ID** | **Primer sequence (5´ - 3´)  Forward/Reverse** | **Amplicon size (bp)** |
| --- | --- | --- | --- | --- |
| - | ^a^18S ribosomal RNA | X51576 | GGGCATTCGTATTTCATAGTCAGA/GTTCTTGATTAATGAAAACATCCT | 98 |
| SltS-1046 | 1-aminocyclopropane-1-carboxylate oxidase | Solyc07g049530.2.1 | ACCATGTCCTAAGCCCGATTT/TGATGCCTCCTGCGTCTGT | 62 |
| SltS-910 | Stress-induced protein sti1-like protein | Solyc08g079170.2.1 | GGCAATTTCACCGACGCTAT/CATGATTGGTGGGAGCAAGA | 64 |
| SltS-117 | Actin depolymerizing factor | Solyc06g005360.2.1 | AAGGGCTTTCCGCTTCATAGT/AACTTTCAGCTGGCTCACCAA | 80 |
| SltS-515 | Inositol monophosphatase 3 | Solyc11g012410.1.1 | CTTCCCCAGCCATAAGTTCATT/TCATCAGTCAGCTCAAAGTTTCCA | 80 |
| SltS-143 | Late embryogenesis abundant protein 2 | Solyc01g095150.2.1 | AACCATGTTAGATGTGCCAGTGA/CGTCCCAATCTCCTCCAATG | 80 |

^a^Reference gene
