# Supplementary material for: Proteometabolomic Study of Compatible Interaction in Tomato Fruit Challenged with Sclerotinia rolfsii Illustrates Novel Protein Network during Disease Progression
Source: Front Plant Sci. 2016 Jul 26;7:1034. doi: 10.3389/fpls.2016.01034 (PMC4960257; doi:10.3389/fpls.2016.01034)
Supplement: Supplementary file 6 [file Table6.DOC]

**Supplementary Table S6.** Multiple sequence alignment of differentially abundant protein paralogues showing amino acid sequence variation. Identical amino acids are depicted by* while conserved and semi-conserved substitutions are marked as : and ., respectively. Dashed line represent gaps inserted for optimal alignment of the sequences.

**(1) ACC oxidase**

....|....| ....|....| ....|....| ....|....| ....|....|

10 20 30 40 50

**SltS-1033** MEN------- ---------- ---------- ---------- ----------

**SltS-1046**  MEN------- ---------- ---------- ---------- ----------

**SltS-826**  MESPRVEESY DKMSELKAFD DTKAGVKGLV DSGITKVPQI FVLPPKDRAK

**Clustal Co** **.

....|....| ....|....| ....|....| ....|....| ....|....|

60 70 80 90 100

**SltS-1033** -------FPI INLEKLNGD- -ERANTMEMI KDACENWGFF ELVNHGIPHE

**SltS-1046**  -------FPI INLEKLNGD- -ERANTMEMI KDACENWGFF ELVNHGIPHE

**SltS-826**  KCETHFVFPV IDLQGIDEDP IKHKEIVDKV RDASEKWGFF QVVNHGIPTS

**Clustal Co** **: *:*: :: * :: : :: : :**.*:**** ::****** .

....|....| ....|....| ....|....| ....|....| ....|....|

110 120 130 140 150

**SltS-1033**  VMDTVEKMTK GHYKKCMEQR --------FK ELVASKGLEA VQAEVTDLDW

**SltS-1046**  VMDTVEKMTK GHYKKCMEQR --------FK ELVASKGLEA VQAEVTDLDW

**SltS-826**  VLDRTLQGTR QFFEQDNEVK KQYYTRDTAK KVVYTSNLDL YKSSVPAASW

**Clustal Co** *:* . : *: .::: * : * ::* :..*: ::.*. .*

....|....| ....|....| ....|....| ....|....| ....|....|

160 170 180 190 200

**SltS-1033_** ESTFFLRHLP TSNISQVPDL DEEYREVMRD FAKRLEKLAE ELLDLLCENL

**SltS-1046**  ESTFFLRHLP TSNISQVPDL DEEYREVMRD FAKRLEKLAE ELLDLLCENL

**SltS-826**  RDTIFCYMAP NP--PSLQEF PTPCGESLID FSKDVKKLGF TLLELLSEGL

**Clustal Co** ..*:* * .. ..: :: * : * *:* ::**. **:**.*.*

....|....| ....|....| ....|....| ....|....| ....|....|

210 220 230 240 250

**SltS-1033_** GLEKGYLKNA FYGSKGPNFG TKVSNYPPCP KPDLIKGLRA HTDAGGIILL

**SltS-1046**  GLEKGYLKNA FYGSKGPNFG TKVSNYPPCP KPDLIKGLRA HTDAGGIILL

**SltS-826**  GLDRSYLKDY MD---CFHLF CSCNYYPPCP QPELTMGTIQ HTDIGFVTIL

**Clustal Co** **::.***: : :: . . ***** :*:* * *** * : :*

....|....| ....|....| ....|....| ....|....| ....|....|

260 270 280 290 300

**SltS-1033_** FQDDKVSGLQ LLKDEQWIDV PPMRHSIVVN LGDQLEVITN GKYKSVLHRV

**SltS-1046**  FQDDKVSGLQ LLKDEQWIDV PPMRHSIVVN LGDQLEVITN GKYKSVLHRV

**SltS-826**  LQDD-MGGLQ VLHQNHWVDV PPTPGSLVVN IGDFLQLLSN DKYLSVEHRA

**Clustal Co** :*** :.*** :*::::*:** ** *:*** :** *::::* .** ** **.

....|....| ....|....| ....|....| ....|....| ....|....|

310 320 330 340 350

**SltS-1033_** IAQTDGTRMS LASFYNPGSD AVIYPAKTLV EKEAEESTQV YPKFVFDDYM

**SltS-1046**  IAQTDGTRMS LASFYNPGSD AVIYPAKTLV EKEAEESTQV YPKFVFDDYM

**SltS-826**  ISNNVGSRMS ITCFFGESPY QSSKLYGPIT ELLSEDNPPK YRATTVKDHT

**Clustal Co** *::. *:*** ::.*:. .. .:. * :*:.. * ...*:

....|....| ....|....| ....|....

360 370

**SltS-1033_** KLYAGLKFQA KEPRFEAMKA MESDPIASA

**SltS-1046**  KLYAGLKFQA KEPRFEAMKA MESDPIASA

**SltS-826**  SYLHNRGLDG -------TSA LSRYKI---

**Clustal Co** . . ::. .* :. *

**(3) Chaperone DnaK**

....|....| ....|....| ....|....| ....|....| ....|....|

10 20 30 40 50

**SltS-498**  MASSTAQIHA LGATYFANSS SSTRKPLKSV FLGQKLNNRT LAFGLKQKKS

**SltS-560**  MASSATHQVK IP---FSGHK LDNRTPFSGG RIS--FASRK KCYSDKAVRQ

**Clustal Co** ****::: : *:. . ..*.*:.. :. : .*. .:. * :.

....|....| ....|....| ....|....| ....|....| ....|....|

60 70 80 90 100

**SltS-498**  RGNNGGYAPM RVVAEKVVGI DLGTTNSAVA AMEGGKPTIV TNAEGQRTTP

**SltS-560**  R-----FRPM KVVNEKVVGI DLGTTNSAVA VMEGGKPTIV TNAEGQRTTP

**Clustal Co** * : ** :** ****** ********** .********* **********

....|....| ....|....| ....|....| ....|....| ....|....|

110 120 130 140 150

**SltS-498**  SVVAYTKSGD RLVGQIAKRQ AVVNPENTFF SVKRFIGRKM NEVDEESKQV

**SltS-560**  SVVAYTKNGD RLVGQIAKRQ SVVNPENTFF SVKRFIGRKM AEVDEESKQV

**Clustal Co** *******.** ********** :********* ********** *********

....|....| ....|....| ....|....| ....|....| ....|....|

160 170 180 190 200

**SltS-498**  SYNVIRDENG NVKLDCPAIG KSFAAEEISA QVLRKLVDDA SKFLNDKVSK

**SltS-560**  SYRVMKDENG NVKLECPAIG KQFAPEEISA QVLRKLVDDA SKFLNDKVAK

**Clustal Co** **.*::**** ****:***** *.**.***** ********** ********:*

....|....| ....|....| ....|....| ....|....| ....|....|

210 220 230 240 250

**SltS-498**  AVVTVPAYFN DSQRTATKDA GRIAGLEVLR IINEPTAASL AYGFEKKSNE

**SltS-560**  AVVTVPAYFN DSQRTATKDA GRIAGLDVLR IINEPTAASL AYGFEKKSNE

**Clustal Co** ********** ********** ******:*** ********** **********

....|....| ....|....| ....|....| ....|....| ....|....|

260 270 280 290 300

**SltS-498**  TILVFDLGGG TFDVSVLEVG DGVFEVLSTS GDTHLGGDDF DKRIVDWLAA

**SltS-560**  TILVFDLGGG TFDVSVLEVG DGVFEVLSTS GDTHLGGDDF DKRIVDWLAD

**Clustal Co** ********** ********** ********** ********** *********

....|....| ....|....| ....|....| ....|....| ....|....|

310 320 330 340 350

**SltS-498**  SFKRDEGIDL LKDKQALQRL TETAEKAKME LSSLTQTNIS LPFITATADG

**SltS-560**  TFRKEEGIEL LKDKQALQRL TEAAEKAKIE LSTLTQTNIS LPFITATADG

**Clustal Co** :*:::***:* ********** **:*****:* **:******* **********

....|....| ....|....| ....|....| ....|....| ....|....|

360 370 380 390 400

**SltS-498**  PKHIETTITR GKFEELCSDL LDRLKTPVQN SLRDAKLSFS DIDEVILVGG

**SltS-560**  PKHIDTTFTR AKFEELCSDL LDRLKTPVET ALKDASLSFK DIDEVVLVGG

**Clustal Co** ****:**:** .********* ********:. :*:**.***. *****:****

....|....| ....|....| ....|....| ....|....| ....|....|

410 420 430 440 450

**SltS-498**  STRIPAVQEL VKKLTGKDPN VTVNPDEVVA LGAAVQAGVL AGDVSDIVLL

**SltS-560**  STRIPAVQNL VRKMTGKEPN VSVNPDEVVA LGASVQAGIL AGDVSDIVLL

**Clustal Co** ********:* *:*:***:** *:******** ***:****:* **********

....|....| ....|....| ....|....| ....|....| ....|....|

460 470 480 490 500

**SltS-498**  DVTPLSIGLE TLGGVMTKII PRNTTLPTSK SEVFSTAADG QTSVEINVLQ

**SltS-560**  DVTPLSLGLE TLGGVMTKII PRNTTLPTSK SEVFSTAADG QTSVEINVLQ

**Clustal Co** ******:*** ********** ********** ********** **********

....|....| ....|....| ....|....| ....|....| ....|....|

510 520 530 540 550

**SltS-498**  GEREFVRDNK SLGSFRLDGI PPAPRGVPQI EVKFDIDANG ILSVTAIDKG

**SltS-560**  GEREFVKDNK SIGRFRLDGI PPAPRGVPQI EVKFDIDTNG ILSVTATDKG

**Clustal Co** ******:*** *:* ****** ********** *******:** ****** ***

....|....| ....|....| ....|....| ....|....| ....|....|

560 570 580 590 600

**SltS-498**  TGKKQDITIT GASTLPGDEV ERMVKEAERF AQEDKEKRDA IDTKNQADSV

**SltS-560**  TGKKQDITIT GASTLPKDEV DRMVQEAEKF AREDKEKREA IDAKNQAESV

**Clustal Co** ********** ****** *** :***:***:* *:******:* **:****:**

....|....| ....|....| ....|....| ....|....| ....|....|

610 620 630 640 650

**SltS-498**  VYQTEKQLKE LGDKVPGPVK EKVEAKLGEL KEAISGGSTQ TMKDAMAALN

**SltS-560**  VYQTEKQLKE LGDKVPADVK NKVESKLKEL KDAISGDSTQ TIKVAMAALN

**Clustal Co** ********** ******. ** :***:** ** *:****.*** *:* ******

....|....| ....|....| ....|....| ....|....| ....|....|

660 670 680 690 700

**SltS-498**  QEVMQLGQSL YNQPGAAPGA GPAPGGADGP SESSSGKGPD GNDVIDADFT

**SltS-560**  QEVMQLGQSL YSQPGPA-GS GPSPGAGTTG SSGSTGKDDG DGEVIDADFS

**Clustal Co** ********** *.***.* *: **:**.. *..*:**. . ..:******:

...

**SltS-498**  DSK

**SltS-560**  ESN

**Clustal Co** :*:

**(4) Chaperonin**

....|....| ....|....| ....|....| ....|....| ....|....|

10 20 30 40 50

**SltS-557**  MASTFAGMSS AGPLAAPSTS S-NKLSSVAN ISSTSFGSKR NVALKKSRRP

**SltS-213**  MYRFAANLAS K--------- -----ASVAR TSSQKIGGRL N--------W

**SltS-625**  ---------- ---------- -----MASTQ LTASSISGNG ----------

**Clustal Co** :. . :...

....|....| ....|....| ....|....| ....|....| ....|....|

60 70 80 90 100

**SltS-557**  TILAAAKELH FNKDGSAIKK LQNGVNKLAD LVGVTLGPKG RNVVLESKYG

**SltS-213**  SRNYAAKDIR FGV--EARAL MLQGVEQLAD AVKVTMGPKG RNVVIEQSWG

**SltS-625**  ---------- ---------- -------FAS FEGLRS---- ----------

**Clustal Co** :*. :

....|....| ....|....| ....|....| ....|....| ....|....|

110 120 130 140 150

**SltS-557**  APKIVNDGVT VAREVELEDP VENIGAKLVR QAAAKTNDLA GDGTTTSVVL

**SltS-213**  APKVTKDGVT VAKSIEFKDK IQNVGASLVK QVANATNDVA GDGTTCATVL

**SltS-625**  --------TC IVKTVSFAPL KHNNSRSFSR LVVKAATTVA PKYTTLKPLG

**Clustal Co** . :.: :.: .* . : : .. :. * . ** :

....|....| ....|....| ....|....| ....|....| ....|....|

160 170 180 190 200

**SltS-557**  AQGLIAEGVK VVAAGANPVL ITRGIEKTAK ALVAELKNMS KEVED-SELA

**SltS-213**  TRAIFAEGCK SVAAGMNAMD LRRGITMAVD SVVTNLKSRA RMISTSEEIA

**SltS-625**  DRVLVK---- ---------- IKTAEEKTVG GILLPVSVQS KPNGG-----

**Clustal Co** : :. : . :. .:: :. : :

....|....| ....|....| ....|....| ....|....| ....|....|

210 220 230 240 250

**SltS-557**  DVAAVSAGNN LEVGSMIAEA MSKVGRKGVV TLEEGKSAEN SLRVVEGMQF

**SltS-213**  QVGTISANGE RVIGDLIARA MEKVGKEGVI TIQDGKTLLN ELDVVEGMKL

**SltS-625**  --EVVAVGEG HSAGKTKVDI SVKTGAQVIY SKYAGTEVEF DG--------

**Clustal Co** ::.. * . *.* . : : ..

....|....| ....|....| ....|....| ....|....| ....|....|

260 270 280 290 300

**SltS-557**  DRGYVSPYFV TDSEKMSVEY ENCKLLLVDK KITNARDLVN VLEDAIRNGY

**SltS-213**  DRGYISPYFI TNQKNQKCEL DNPLILIHEK KISSINAVVK ALELALKRQR

**SltS-625**  ---------- ---------- -SKHLILKED DIVGILETDD VKDLQPLNDR

**Clustal Co** . ::: :. .* :

....|....| ....|....| ....|....| ....|....| ....|....|

310 320 330 340 350

**SltS-557**  PILIIAEDIE QEALATLVVN KLRGALKVAA LKAPGFGERK SQYLDDIATL

**SltS-213**  PLLIVAEDVD NEALATLILN KLRAGIKVCA IKAPGFGENR KAYLQDLAIL

**SltS-625**  VLIKVAEAEE KTAGGLLLTE AAKEKPSIGT IIAVGPG--- ----------

**Clustal Co** :: :** * . *: : : .: : : * * *

....|....| ....|....| ....|....| ....|....| ....|....|

360 370 380 390 400

**SltS-557**  TGGTVIREEL GLTLDKADKE VLGHAAKVVL TKDATTIVGD GSTQEAVNKR

**SltS-213**  TGGQVITEEL GLNIENLEFE MLGTSKEATI SKDDTVILDG AGEKKSIEER

**SltS-625**  ---------- -------PLD EEGNRKPLSV SPGNTVLYSK YAG-------

**Clustal Co** : * : : . *.: .

....|....| ....|....| ....|....| ....|....| ....|....|

410 420 430 440 450

**SltS-557**  VAQIKNLIEA ADQDYEKEKL NERIAKLSGG VAVIQVGAQT ETELKEKKLR

**SltS-213**  CELIRSTIEQ STSDYDKEKL QERLAKLSGG VAVLKIGGAS EAEVGEKKDR

**SltS-625**  ----SEFKGA DGSDYITLRV SDVMAVLS-- ---------- ----------

**Clustal Co** . . * . :: : :* **

....|....| ....|....| ....|....| ....|....| ....|....|

460 470 480 490 500

**SltS-557**  VEDALNATKA AVEEGIVVGG GCTLLRLAAK VDAIKGTLAN DEEKVGADIV

**SltS-213**  VTDALNATKA AVEEGIVPGG GVALLYAARE LDNLTT--AN FDQKIGVQII

**SltS-625**  ---------- ---------- ---------- ---------- ----------

**Clustal Co**

....|....| ....|....| ....|....| ....|....| ....|....|

510 520 530 540 550

**SltS-557**  KRALSYPLKL IAKNAGVNGS VVSEKVLSSD DPKFGYNAAT GNYEDLMAAG

**SltS-213**  QNALKTPVHT IASNAGVEGA VVVGKLLDQD NLDLGYDAAK GEYVDMIKAG

**SltS-625**  ---------- ---------- ---------- ---------- ----------

**Clustal Co**

....|....| ....|....| ....|....| ....|....| ....|....|

560 570 580 590 600

**SltS-557**  IIDPTKVVRC CLEHAASVAK TFLMSDCVVV EIKEPEAAVA GNPMDNSGYG

**SltS-213**  IIDPVKVIRT ALVDAASVSS LLTTTEAVVV ELPKDEKESP AMGGGMGGGM

**SltS-625**  ---------- ---------- ---------- ---------- ----------

**Clustal Co**

....|

**SltS-557**  Y----

**SltS-213**  GGMDF

**SltS-625**  -----

**Clustal Co**

**(6) Late Embryogenesis Protein**

....|....| ....|....| ....|....| ....|....| ....|....|

10 20 30 40 50

**SltS-143**  MMSSSENPEI VERVFKDKDE EEKDEQKGSF IDKVKGFIQD IGEKIEETVG

**SltS-284**  MMSSSENPEI VERVFKDKDE EEKDEQKGSF IDKVKGFIQD IGEKIEETVG

**Clustal Co** .: ***.*.*:. *: ..

....|....| ....|....| ....|....| ....|....| ....|....|

60 70 80 90 100

**SltS-143**  FGKPTADVSG IHIPHINLEK AEIVVDVLVK NPNPIPIPLI DINYLIESDG

**SltS-284**  FGKPTADVSG IHIPHINLEK AEIVVDVLVK NPNPIPIPLI DINYLIESDG

**Clustal Co** : ** * ::. :.: *.:: : ..* ** ** .:***:: :*:*:::*

....|....| ....|....| ....|....| ....|....| ....|....|

110 120 130 140 150

**SltS-143**  RELLSGLIPD AGTIHAHGSE TVKIPLNLVY DDIRTTYHDI KPGSIIPYKI

**SltS-284**  RELLSGLIPD AGTIHAHGSE TVKIPLNLVY DDIRTTYHDI KPGSIIPYKI

**Clustal Co** * : ** *** .*:*:*:.* :.:*::: : . : : :** . : *.:

....|....| ....|....| ....|....| ....|....| ....|....|

160 170 180 190 200

**SltS-143**  KVDLIVDVPV FGRITIPLEK NGEI------ ---------- ----------

**SltS-284**  KVDLIVDVPV FGRITIPLEK NGEI------ ---------- ----------

**Clustal Co** ::.**:*:** :*.*****. .**

....|..

**SltS-143**  -------

**SltS-284**  -------

**Clustal Co**

**(7) Malate dehydrogenase**

....|....| ....|....| ....|....| ....|....| ....|....|

10 20 30 40 50

**SltS-516**  MAATSATTLS VGSTTSLGCK GSSISQSKAF GVKFNSKNNI RSFSGLKAAT

**SltS-897**  ---------- ---------- ---------- ---------- ----MAKDPV

**SltS-1093**  ---------- ---------- ---------- ---------- ----MAKDPV

**Clustal Co** * .

....|....| ....|....| ....|....| ....|....| ....|....|

60 70 80 90 100

**SltS-516**  TVSCESESSF IGKESLAALK QSITPKAQKG NRGYVSCVQP QASYKVAILG

**SltS-897**  RVLVTGAAGQ IGY------- ---------- ---------- -ALVPMIARG

**SltS-1093**  RVLVTGAAGQ IGY------- ---------- ---------- -ALVPMIARG

**Clustal Co** . :. :. : *

....|....| ....|....| ....|....| ....|....| ....|....|

110 120 130 140 150

**SltS-516**  ASGGIGQPLA LLVKMSPLVS ELNLYDIANV KGVAADLSHC NTPSQVSDFT

**SltS-897**  VMLGADQPVI LHMLDIPPAA EA-------L NGVKMELVDA AFP-LLKGVV

**SltS-1093**  VMLGADQPVI LHMLDIPPAA EA-------L NGVKMELVDA AFP-LLKGVV

**Clustal Co** . * .**: * : * .: . ** :: . : ...

....|....| ....|....| ....|....| ....|....| ....|....|

160 170 180 190 200

**SltS-516**  GASELANCLK GVNVVVIPAG VPRKPGMTRD DLFNINANIV KTLVEAVADN

**SltS-897**  ATTDAVEACT GVNVAVMVGG FPRKEGMERK DVMSKNVSIY KSQASALEKH

**SltS-1093**  ATTDAVEACT GVNVAVMVGG FPRKEGMERK DVMSKNVSIY KSQASALEKH

**Clustal Co** . : :. *.:*.:: .* .*** ** *. *::. *..* *: *: .

....|....| ....|....| ....|....| ....|....| ....|....|

210 220 230 240 250

**SltS-516**  -CPDAFIHII SNPVNSTVPI AAEVLKRKGV YDPKKLFGVT TLDVVRANTF

**SltS-897**  AAPNCKVLVV ANPANTNALI LKEFAP---S IPEKNITCLT RLDHNRALGQ

**SltS-1093**  AAPNCKVLVV ANPANTNALI LKEFAP---S IPEKNITCLT RLDHNRALGQ

**Clustal Co** .*:. : :: :**.*:.. * *. *:: :* ** **

....|....| ....|....| ....|....| ....|....| ....|....|

260 270 280 290 300

**SltS-516**  VAQKKNLRLI DVDVPVVGGH AGITILPLLS KTKPSTTFTD EEVQELTVRI

**SltS-897**  ISERLSVQVS DVKNVIIWGN HSSSQYPDVN HATVSTPAGD KPVRELVADD

**SltS-1093**  ISERLSVQVS DVKNVIIWGN HSSSQYPDVN HATVSTPAGD KPVRELVADD

**Clustal Co** : : .:.: :*. :: *: . : * .. ::. .: * : : *.

....|....| ....|....| ....|....| ....|....| ....|....|

310 320 330 340 350

**SltS-516**  QNAGTEVVEA KAGAGSATLS ---MAYAAAR FVESSLRALD GDSDVYECAF

**SltS-897**  AWLNGEFIST VQQRGAAIIK ARKLSSALSA ASSACDHIRD WVLGTPEGTF

**SltS-1093**  AWLNGEFIST VQQRGAAIIK ARKLSSALSA ASSACDHIRD WVLGTPEGTF

**Clustal Co** . *.:.: *:* :. :: * : .:. : : .. * :*

....|....| ....|....| ....|....| ....|....| ....|....|

360 370 380 390 400

**SltS-516**  VQSDIS---- ---ELPFFAS RIKIGKNGVE ALISSDLQGL SEYEQKALDA

**SltS-897**  VSMGVYSDGS YNVPAGLIYS FPVTCKNGEW SIVQG--LPI DEFSRKKLDL

**SltS-1093**  VSMGVYSDGS YNVPAGLIYS FPVTCKNGEW SIVQG--LPI DEFSRKKLDL

**Clustal Co** *. .: :: * *** :: : .::.:: *:

....|....| ....|....| ...

410 420

**SltS-516**  LKPELKSSIE KGTGFVQKEP VAA

**SltS-897**  TAEELSE--E KALAYSCLA- ---

**SltS-1093**  TAEELSE--E KALAYSCLA- ---

**Clustal Co** ** . * *. :
