# Supplementary material for: Proteometabolomic Study of Compatible Interaction in Tomato Fruit Challenged with Sclerotinia rolfsii Illustrates Novel Protein Network during Disease Progression
Source: Front Plant Sci. 2016 Jul 26;7:1034. doi: 10.3389/fpls.2016.01034 (PMC4960257; doi:10.3389/fpls.2016.01034)
Supplement: Supplementary file 7 [file Table7.DOCX]

**Supplementary Table S7**. Metabolite composition of tomato fruits challenged with *Sclerotinia*.

| **Category** | **Metabolite** | **Untreated**  **(Relative response ration)** | **SD (±)** | **Treated**  **(Relative response ration)** | **SD (±)** |
| --- | --- | --- | --- | --- | --- |
| Aminoacid | L-Tryptophan | 11.149 | 1.242 | 1.004 | 0.024 |
|  | L-Lysine | 1.206 | 0.084 | 13.162 | 0.490 |
|  | Glycine | 1.120 | 0.140 | 2.430 | 0.024 |
|  | l-Isoleucine | 2.624 | 0.335 | 2.464 | 0.055 |
|  | l-Leucine | 0.753 | 0.099 | 1.364 | 0.030 |
|  | L-Norleucine | 0.525 | 0.091 | 8.149 | 0.169 |
|  | L-Norvaline | 0.158 | 0.034 | 7.413 | 0.249 |
|  | valyl- valine | 2.335 | 0.067 | 2.324 | 0.004 |
|  | Methyl -isoleucine | 3.451 | 0.077 | 1.231 | 0.003 |
|  | Citrulline | 2.121 | 0.012 | 2.561 | 0.012 |
|  | bis(2-hydroxypropyl)amine | 2.341 | 0.213 | 2.325 | 0.213 |
|  | Ornithine | 0.451 | 0.004 | 0.541 | 0.003 |
| Carbohydrate | Dihydroxyacetone | 0.420 | 0.028 | 2.664 | 0.037 |
|  | D-Glucose | 335.147 | 13.386 | 106.516 | 2.215 |
|  | D-Glyceraldehyde | 2.158 | 0.170 | 8.766 | 0.882 |
|  | gluconic lactone | 0.322 | 0.019 | 0.008 | 0.002 |
|  | N-acetyl-beta-D-mannosamine | 3.121 | 0.043 | 12.325 | 0.062 |
|  | L-threose | 1.324 | 0.002 | 1.431 | 0.011 |
|  | Raffinose | 0.221 | 0.002 | 0.312 | 0.006 |
| Caretenoids | Mevalonic acid | 1.213 | 0.003 | 2.221 | 0.033 |
| Fatty acid | alpha.-Linolenic acid | 14.322 | 0.910 | 10.828 | 0.114 |
|  | Hexadecanoic acid | 4.758 | 0.908 | 16.910 | 0.400 |
|  | Palmatoleic acid | 2.769 | 0.359 | 0.093 | 0.007 |
| Flavanone | Naringenin | 0.255 | 0.018 | 0.257 | 0.001 |
| Indole | 3-indoleacetonitrile | 1.021 | 0.101 | 1.221 | 0.003 |
| Nucleobase/ Nucleotide | Uridine | 0.221 | 0.010 | 0.008 | 0.001 |
|  | Adenine | 0.251 | 0.012 | 0.008 | 0.003 |
|  | 5,6-dihydrouracil | 0.324 | 0.020 | 0.009 | 0.006 |
| Organic/ inorganic acid | Phosphoric acid | 53.050 | 3.604 | 21.359 | 0.457 |
|  | Pyruvic acid | 2.398 | 0.232 | 6.092 | 0.025 |
|  | Malic acid | 0.339 | 0.069 | 0.088 | 0.003 |
|  | Oxaloacetic acid | 0.651 | 0.186 | 0.072 | 0.003 |
|  | Citric acid | 0.506 | 0.018 | 0.301 | 0.044 |
|  | Oxalic acid | 3.006 | 0.391 | 8.050 | 0.114 |
|  | Gluconic acid | 4.271 | 0.419 | 6.684 | 0.147 |
|  | L-Ascorbic acid | 6.016 | 1.155 | 5.204 | 0.103 |
|  | Acetic acid | 0.249 | 0.017 | 0.016 | 0.004 |
|  | Mannonic acid | 1.892 | 0.034 | 3.121 | 0.143 |
|  | Erythropentonic acid | 1.612 | 0.051 | 3.012 | 0.122 |
|  | 3,4 Dihydroxymandelic acid | 2.134 | 0.112 | 0.567 | 0.001 |
|  | Methylmalonic acid | 2.023 | 0.001 | 2.512 | 0.112 |
|  | Shikimic acid | 1.221 | 0.341 | 1.252 | 0.113 |
|  | Maleamate | 0.522 | 0.003 | 0.646 | 0.001 |
|  | Caproic anhydride | 0.113 | 0.003 | 0.117 | 0.002 |
| Phenolics | Caffeic acid | 0.337 | 0.020 | 0.009 | 0.002 |
|  | 4-hydroxy-6-methyl-2-pyrone | 0.114 | 0.000 | 0.141 | 0.221 |
| Quinazoline | 4-hydroxyquinazoline | 2.321 | 0.023 | 1.092 | 0.004 |
| Sugar alcohol | D-Mannitol | 2.073 | 0.134 | 2.095 | 0.049 |
|  | Glycerol | 1.377 | 0.114 | 0.393 | 0.067 |
|  | Pinitol | 4.121 | 0.213 | 11.214 | 0.109 |
|  | Xylitol | 2.324 | 0.111 | 10.332 | 0.111 |
|  | myo inositol | 1.245 | 0.045 | 0.113 | 0.002 |
|  | Threitol | 1.121 | 0.115 | 1.282 | 0.003 |
|  | 1,5-anhydroglucitol | 0.222 | 0.005 | 0.226 | 0.004 |
| Thiazole | 4-methyl-5-thiazolethanol | 1.214 | 0.029 | 8.451 | 0.112 |
| Miscelleneous | Hydroxybutane | 0.981 | 0.001 | 1.211 | 0.002 |
|  | Acetanilide | 0.213 | 0.003 | 0.223 | 0.001 |
