# Supplementary material for: Proteometabolomic Study of Compatible Interaction in Tomato Fruit Challenged with Sclerotinia rolfsii Illustrates Novel Protein Network during Disease Progression
Source: Front Plant Sci. 2016 Jul 26;7:1034. doi: 10.3389/fpls.2016.01034 (PMC4960257; doi:10.3389/fpls.2016.01034)
Supplement: Supplementary file 8 [file Presentation1.PDF]

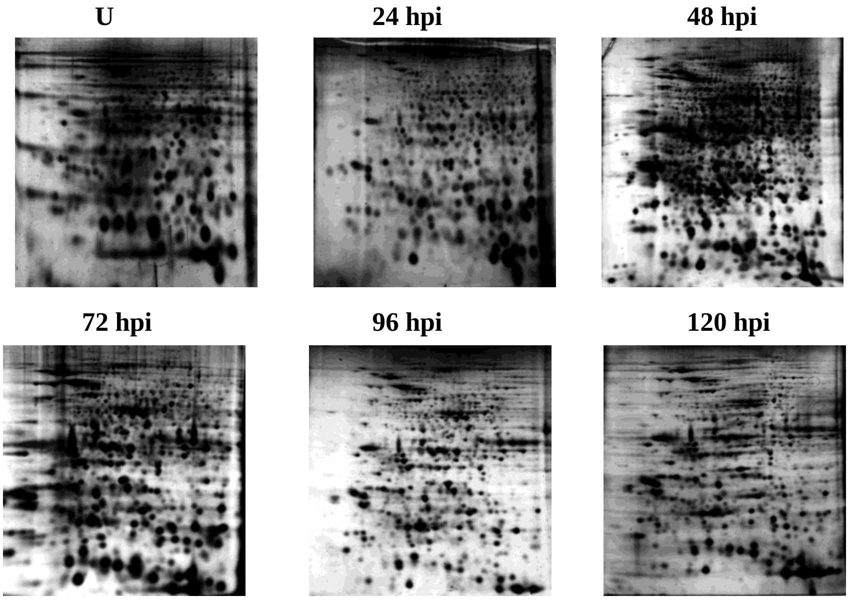

Supplementary Figure S1. Raw gel images of each time points (U and 24-120 hpi.

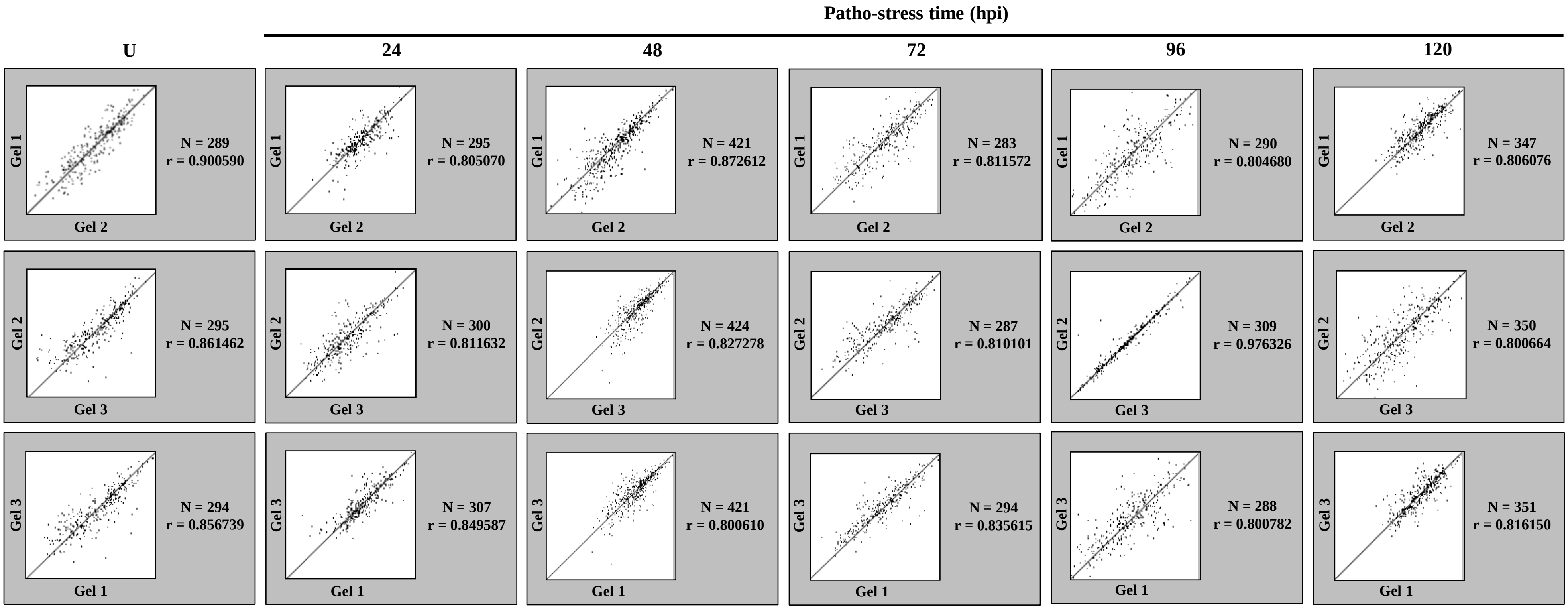

Supplementary Figure S2. Scatter plots displaying a correlation coefficient of variation above 0.8 among the three replicate gels of the patho-stressed as well as unstressed fruits. U, represents unstressed; numbers represent time points; N, stands for number of spots.

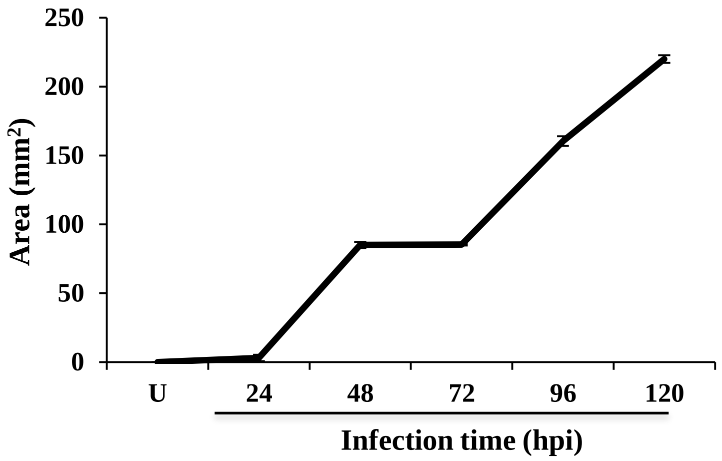

Supplementary Figure S3. Disease severity curve of tomato fruit challenged with *Sclerotinia rolfsii* in time course. U, uninfected.

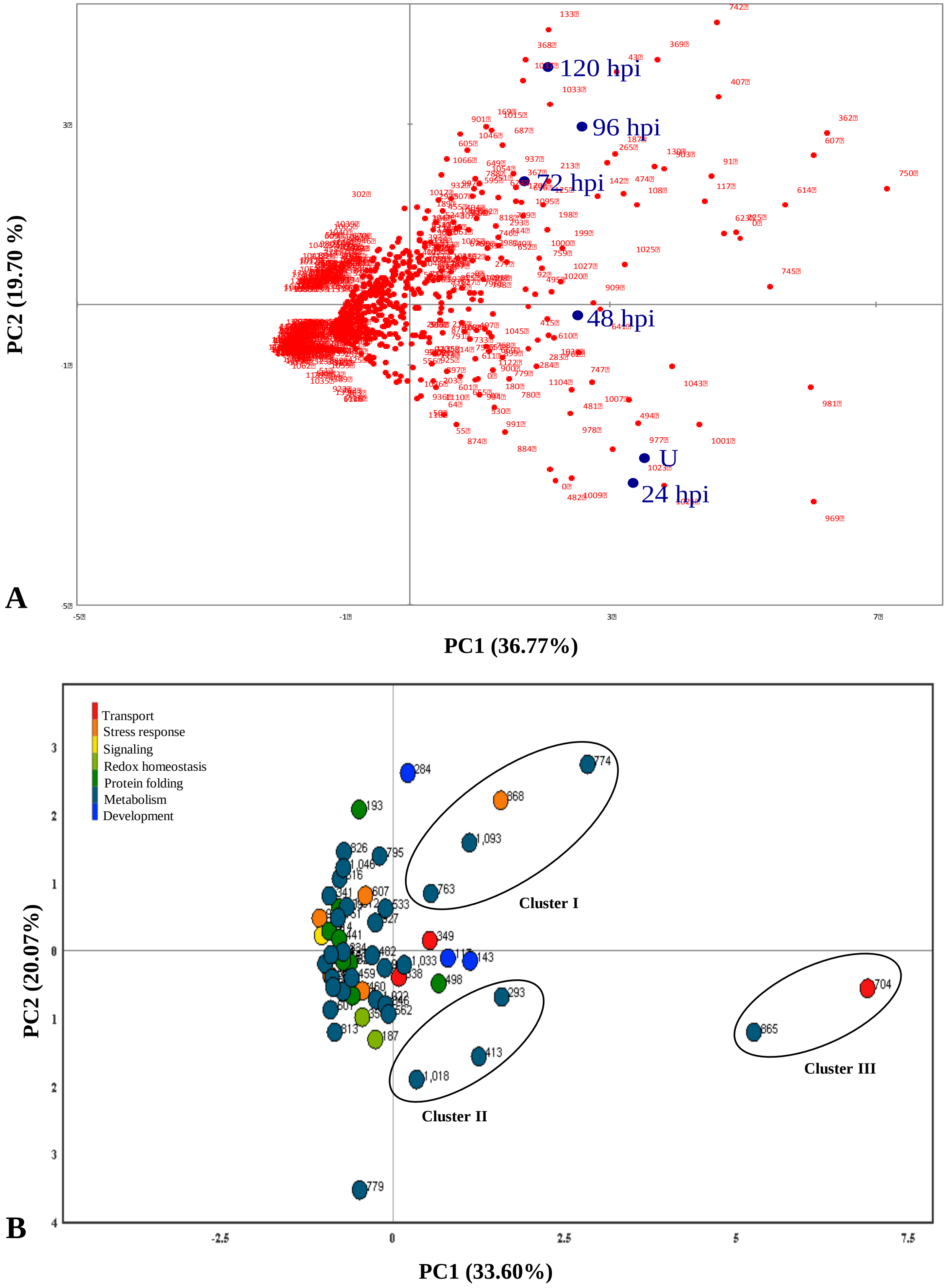

Supplementary Figure S4. Principal component analysis of (A) all the differentially abundant spots, (B) identified proteins. Scree plot, x-axis represents principal component 1 and y-axis represents principal component 2. The color represents functional categories of identified proteins. Circles represent significantly changed protein clusters.

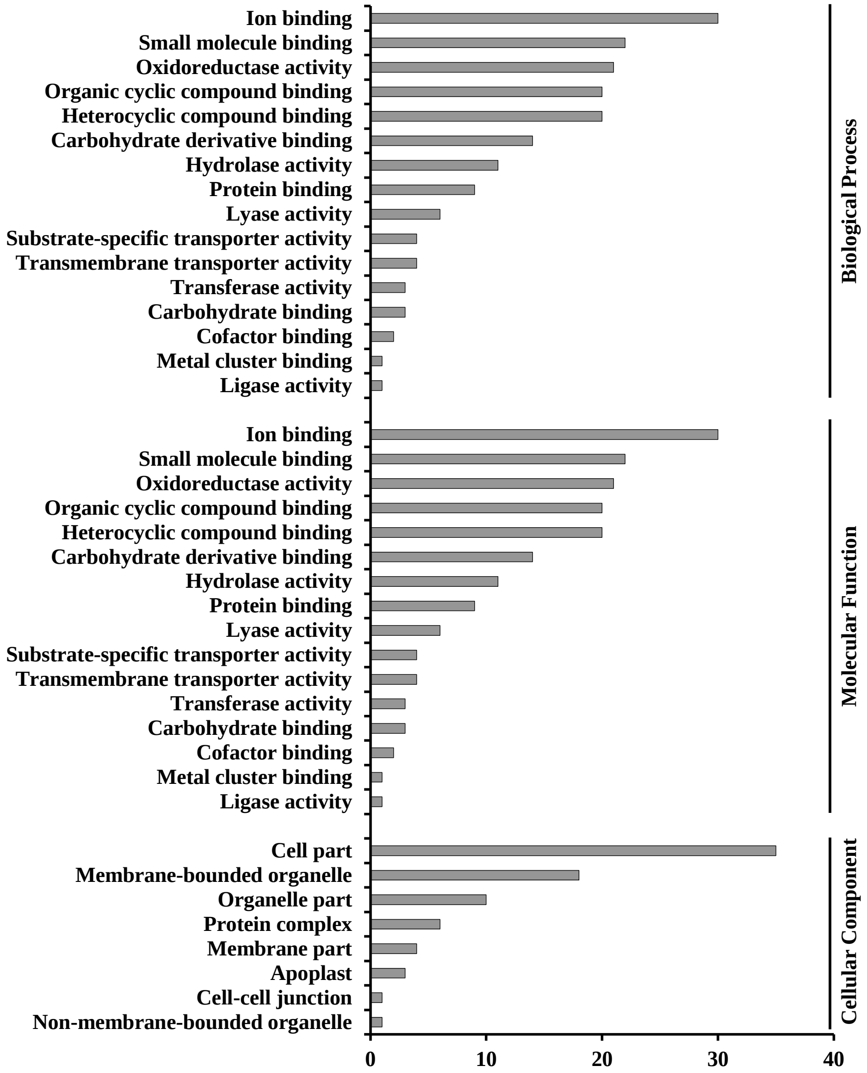

Supplementary Figure S5. Distribution of identified PSRPs according to GO functional category (biological process, molecular function, and cellular component) by Blast2GO. The Y-axis exhibits GO terms and the X-axis shows number of proteins.

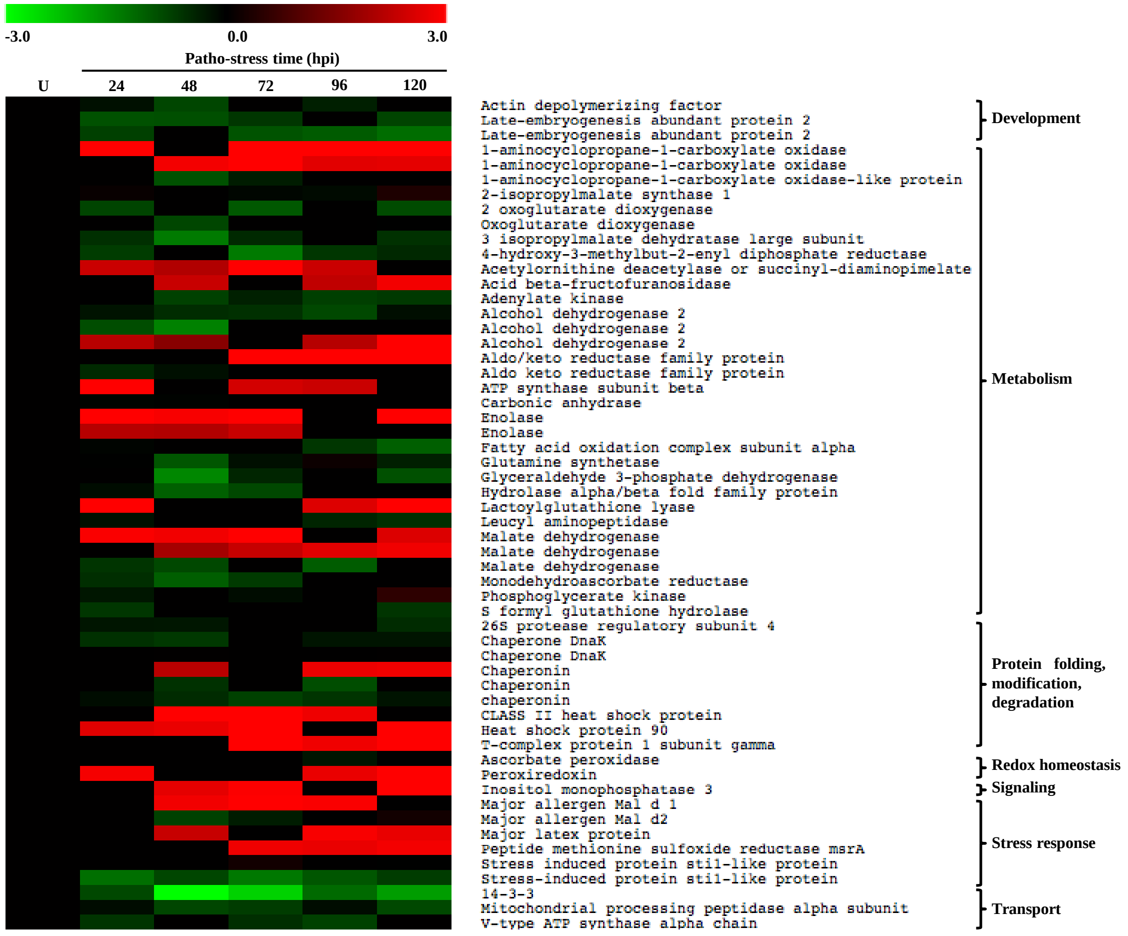

Supplementary Figure S6. Heat map of PSRPs according to functional categories. Each protein is represented by a single row of coloured boxes and each of the unstressed (U) and patho-stressed time points (24, 48, 72, 96, 120 hpi) are represented by a single column. High (or low) abundance ranges from pale to saturated red (or green).
